# Supplementary material for: Dual targeting of tumoral cells and immune microenvironment by blocking the IL-33/IL1RL1 pathway
Source: Nat Commun. 2025 Jul 14;16:6369. doi: 10.1038/s41467-025-61567-7 (PMC12259856; doi:10.1038/s41467-025-61567-7)
Supplement: Supplementary file 1 — Supplementary Information [file 41467_2025_61567_MOESM1_ESM.pdf]

Supplementary Materials for

Dual targeting of tumoral cells and immune microenvironment by blocking the IL-33/IL1RL1 pathway

The PDF file includes:

Supplementary Fig. 1. IL1RL1 expression in healthy donors, AML patients with different cytogenetics, and $CD34^+CD38^-$LSCs in the BM from AML non-responders (NR) and complete responders (CR), and normal HSCs and human $CD34^+CD38^-CD90.2^+CD45RA^-$LSCs.

Supplementary Fig. 2. Gating strategies for flow cytometric analysis.

Supplementary Fig. 3. Cytotoxic T cells and regulatory T cells in nonresponders (NR) vs. complete responders (CR) following chemotherapy induction.

Supplementary Fig. 4. Il1rl1 is indispensable for steady-state hematopoiesis.

Supplementary Fig. 5. Homing potential of WT and $Il1rl1^{-/-}$ BM cells.

Supplementary Fig. 6. Frequencies of GFP^+ leukemic cells and MLL-AF9 leukemic stem cells (LSCs) in the BM of mice from the leukemogenesis initiation model.

Supplementary Fig. 7. Il1rl1 expression in murine HSCs, LSCs, and LPSCs, and Mito Tracker Deep Red of MLL-AF9 leukemic cells.

Supplementary Fig. 8. KEGG pathway analysis of differentially expressed genes between WT and $Il1rl1^{-/-}$ MLL-AF9 LSCs.

Supplementary Fig. 9. Il1rl1 expression in MLL-AF9 leukemic cells in the BM of mice from the leukemia maintenance model before and after pIpC administration.

Supplementary Fig. 10. Frequencies, proliferation, apoptosis, and reactive oxygen species of GFP^+ leukemic cells in the leukemia maintenance model.

Supplementary Fig. 11. Stress induced IL-33/Il1rl1 signaling autocrine loop in normal hematopoietic stem cells.

Supplementary Fig. 12. Leukemia immune microenvironment cell subsets analysis of IL-33/Il1rl1 signaling autocrine loop model.

Supplementary Fig. 13. Frequencies of $CD4^+$, $CD8^+$, and regulatory T cells in non-leukemic wild type and IL-$33^{cit/cit\ KO}$ mice at steady state.

Supplementary Fig. 14. Cell cycle and reactive oxygen species of GFP^+ leukemic cells in IL-33/Il1rl1 signaling autocrine loop model.

Supplementary Fig. 15. Il1rl1 deficiency in MLL-AF9 LSC inhibits their growth.

Supplementary Fig. 16. IL1RL1 deficiency in MOLM14 cells decreases their proliferation and increases apoptosis.

Supplementary Fig. 17. IL1RL1 deficiency inhibits MOLM14 cells growth to extend survival.

Supplementary Fig. 18. Stability and pharmacodynamic study of anti-IL1RL1 T-BsAbs.

Supplementary Fig. 19. Cytotoxicity of anti-mIl1rl1 T-BsAbs on MLL-AF9 cells.

Supplementary Fig. 20. Il1rl1 expression in leukemic cells and LSCs after anti-Il1rl1 T-BsAbs treatment.

Supplementary Fig. 21. Il1rl1 expression in non-LSC progenitors after anti-Il1rl1 T-BsAbs treatment at Day 10.

Supplementary Fig. 22. Absolute numbers of $CD3^+CD8^+$, $WT\text{-}1^+CD8^+$, $CD3^+CD4^+$, and $Il1rl1^+Foxp3^+$T cells, and the $CD8^+$T cells to leukemic cells ratio in BC281- vs. BC462-treated leukemic mice.

Supplementary Fig. 23. Anti-Il1rl1 T-BsAb reverses the immunosuppressive microenvironment.

Supplementary Fig. 24. Il1rl1 expression in leukemic cells and LSCs following anti-Il1rl1 T-BsAb treatment in combination with adoptive transfer of $CD8^+$T cells.

Supplementary Fig. 25. Functional test of ALT-803 *in vivo*.

Supplementary Fig. 26. Il1rl1 expression in leukemic cells and LSCs following anti-Il1rl1 T-BsAb treatment in combination with ALT-803 model.

Supplementary Fig. 27. Dual targeting with anti-Il1rl1 T-BsAb in an epigenetically induced immunocompetent myeloid leukemia model.

Supplementary Fig. 28. *In vitro* cytotoxicity of anti-IL1RL1 T-BsAbs co-cultured with human $CD8^+$ T cells on pediatric-AML PDX cells.

Supplementary Fig. 29. Anti-human IL1RL1 T-BsAb in PDX AML reinduction model.

Supplementary Table 1. AML patients' demographics.

Supplementary Table 2. Expression of leukemia oncogenic drivers comparing RNA-seq in $Il1rl1^{f/f}$ vs $Il1rl1^{f/f\ Mx1Cre}$ LSCs.

Supplementary Table 3. Expression of cell cycle signatures comparing RNA-seq in $Il1rl1^{f/f}$ vs $Il1rl1^{f/f\ Mx1Cre}$ LSCs.

Supplementary Table 4. Expression of metabolomic signatures comparing RNA-seq in $Il1rl1^{/f}$ vs $Il1rl1^{f/f\ Mx1Cre}$ LSCs.

Supplementary Table 5. Literature references for oncogenic drivers, cell cycle and metabolomic signatures derived from comparing RNA-seq in $Il1rl1^{f/f}$ vs $Il1rl1^{f/f\ Mx1Cre}$ LSCs.

Supplementary Table 6. Anti-IL1RL1 neutralizing antibody doesn't not react with normal human tissues.

Supplementary Table 7. Patient demographics and sample characteristics of AML PDX models.

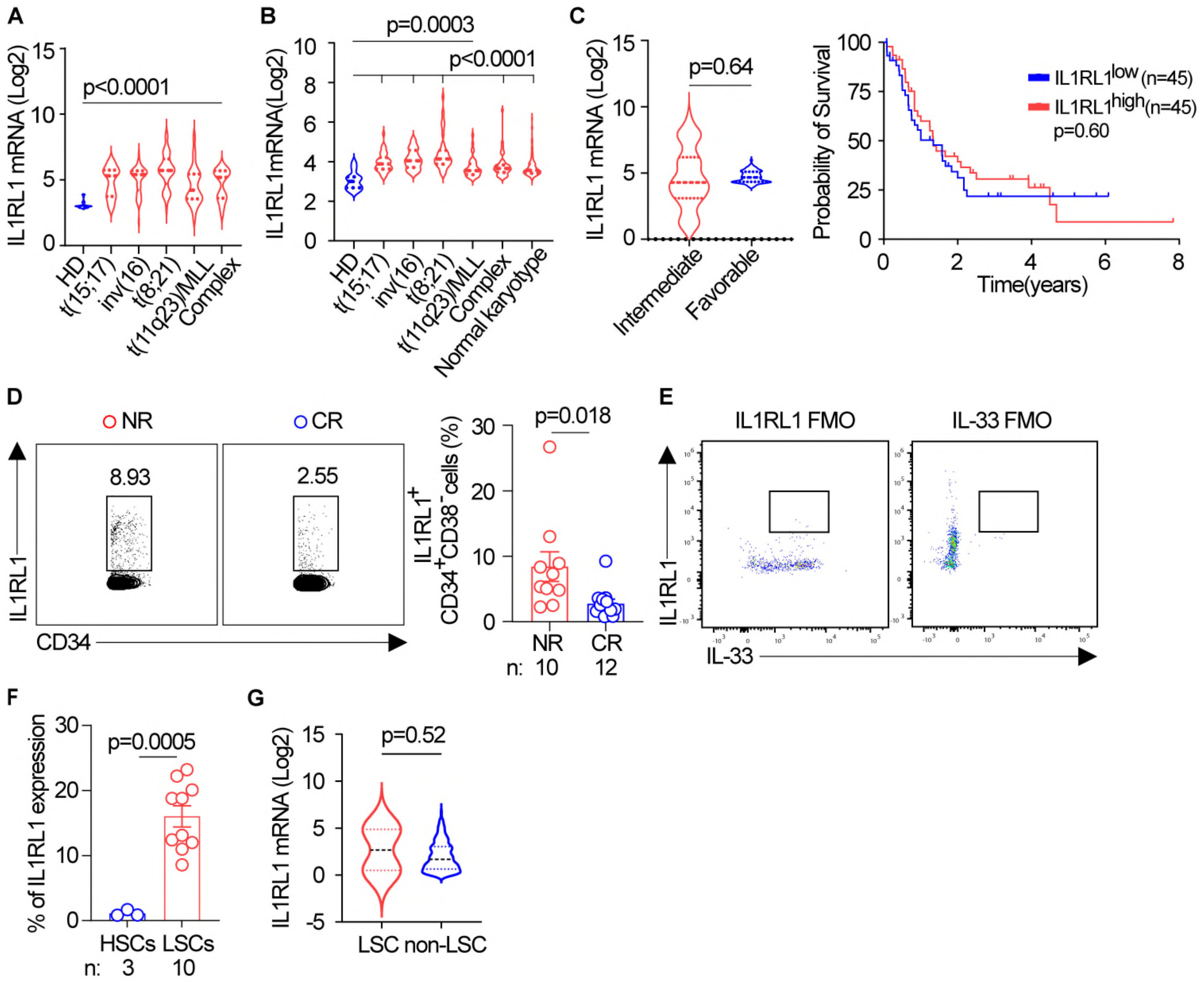

Supplementary Fig. 1. IL1RL1 expression in healthy donors, AML patients with different cytogenetics, and CD34$^+$CD38$^-$LSCs in the BM from AML nonresponders (NR) and complete responders (CR), and normal HSCs and human CD34$^+$CD38$^-$CD90.2$^+$CD45RA$^-$LSCs**.**

(A) IL1RL1 expression in healthy donors (n=46) and AML patients with different cytogenetics (t(15;17), n=39; inv(16), n=53; t(8;21), n=57; t(11q23/MLL), n=27; Complex, n=23). (**B**) IL1RL1 expression in healthy donors (n=16) and AML patients with different cytogenetics (t(15;17), n=37; inv(16), n=28; t(8;21), n=40; t(11q23/MLL), n=38; Complex, n=48; Normal karyotype, n=351). (**C**) IL1RL1 expression in intermediate-risk AML patients (characterized by the absence of favorable or unfavorable cytogenetic and molecular abnormalities, n=161) and favorable-risk AML patients [t(8;21), t(15;17), inv(16)](n=95). Kaplan-Meier survival curve for intermediate-risk AML patients stratified by IL1RL1^{low} and IL1RL1^{high} expression levels. (**D**) Representative flow plots for nonresponders (NR) and complete responders (CR) IL1RL1$^+$CD34$^+$CD38$^-$LSCs (gating on CD45$^+$CD33$^+$) and statistical quantities. Data are mean±s.e.m (n=10 or 12); (**E**) IL1RL1 and IL-33 FMO flow staining in AML patients

with complete responders and non-responders. (**F**) IL1RL1^{+}HSCs from healthy donors (HD) and IL1RL1^{+}LSCs (defined as$CD34^{+}CD38^{-}CD90.2^{+}CD45RA^{-}$) from AML patients calculated by flow cytometry. (**G**) IL1RL1 expression in LSC ($CD34^{+}CD38^{-}$, n=2) and non-LSC leukemic cells (n=363) measured by bulk RNA-sequencing. (**A**) and (**B**), ANOVA was used. (**C**), (**D**), (**F**), and (**G**), unpaired t-test was used. For (**D**) survival analysis was analyzed by log-rank test.

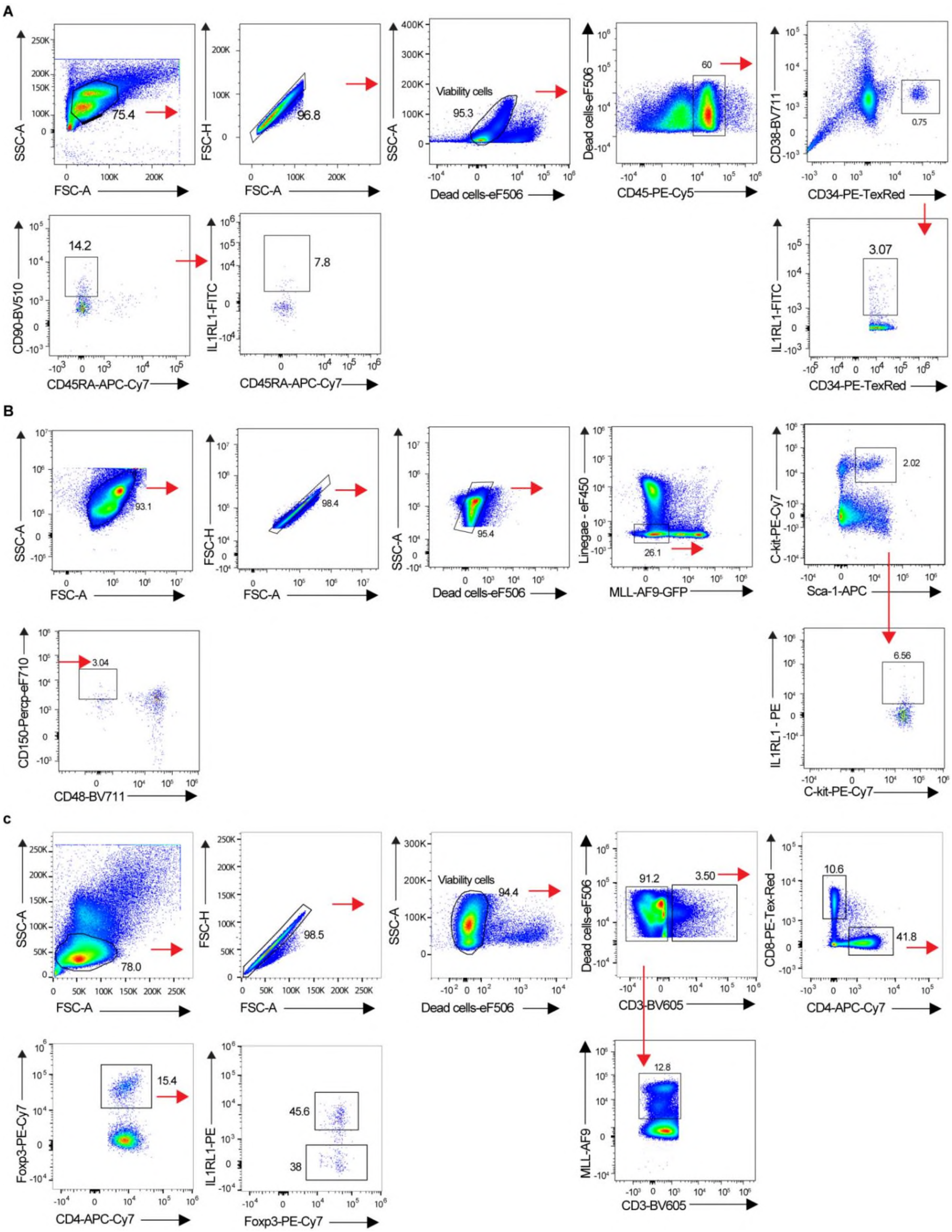

Supplementary Fig. 2. Gating strategies for flow cytometric analysis. (**A**) Gating strategy for IL1RL1$^+$LSCs (CD34$^+$CD38$^-$CD90$^+$CD45RA$^-$) cells from AML patients' samples. (**B**) Gating strategy in murine AML samples for Il1rl1$^+$ LSCs (Lineage$^-$ MLL-AF9 cells – C-kit$^+$Sca-1$^+$ LSCs -CD150$^+$CD48$^-$ long-term LSCs). (**C**) Gating strategy in murine AML samples for Il1rl1$^+$ Treg cells (CD3$^+$T cells - CD4$^+$T cells -CD4$^+$Foxp3$^+$T cells - Il1rl1$^+$Foxp3$^+$T cells and MLL-AF9^{gfp} cells (gated on CD3$^-$ cells).

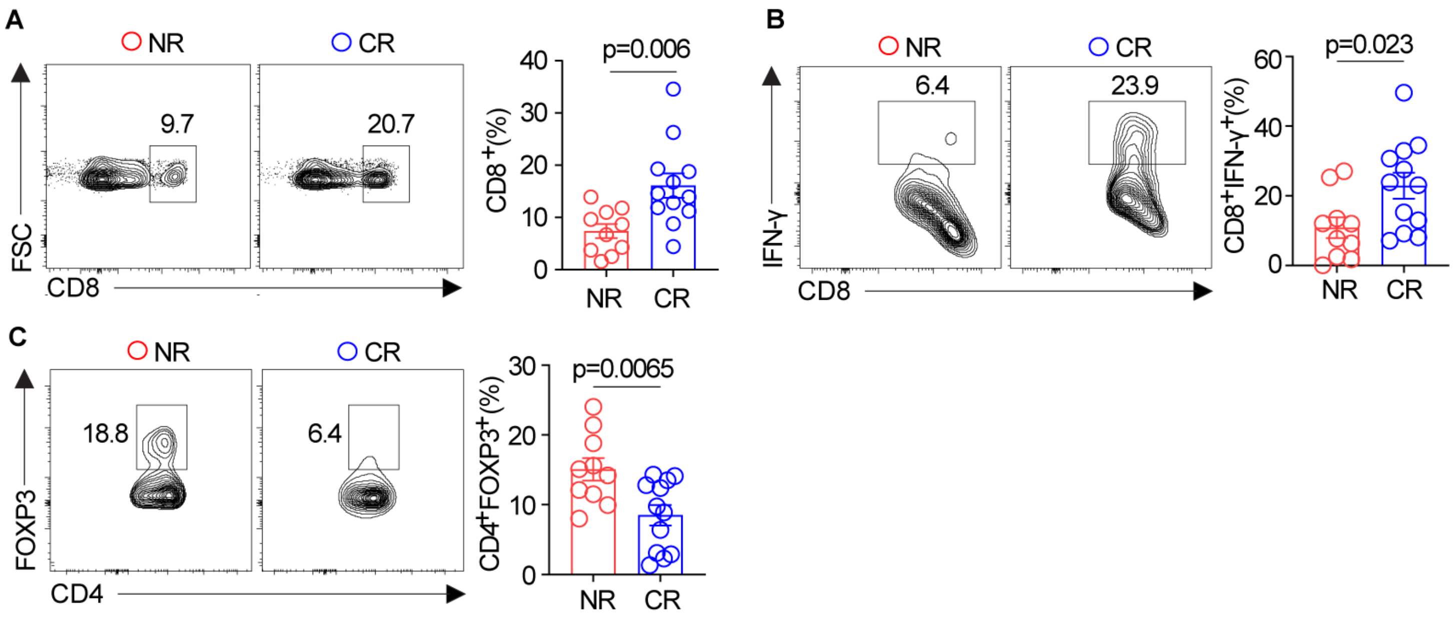

Supplementary Fig.3. Cytotoxic T cells and regulatory T cells in nonresponders and complete responders following chemotherapy induction. (**A**) Frequencies of $CD8^+$ T cells in NR (n=10) and CR (n=12); (**B**) Frequencies of $CD8^+IFN\text{-}\gamma^+$T cells in NR (n=10) and CR (n=12); (**C**) Frequency of $CD4^+FOXP3^+$ regulatory T cells in NR (n=10) and CR (n=12). Data are mean ± s.e.m.; Unpaired two-sided t-test was used.

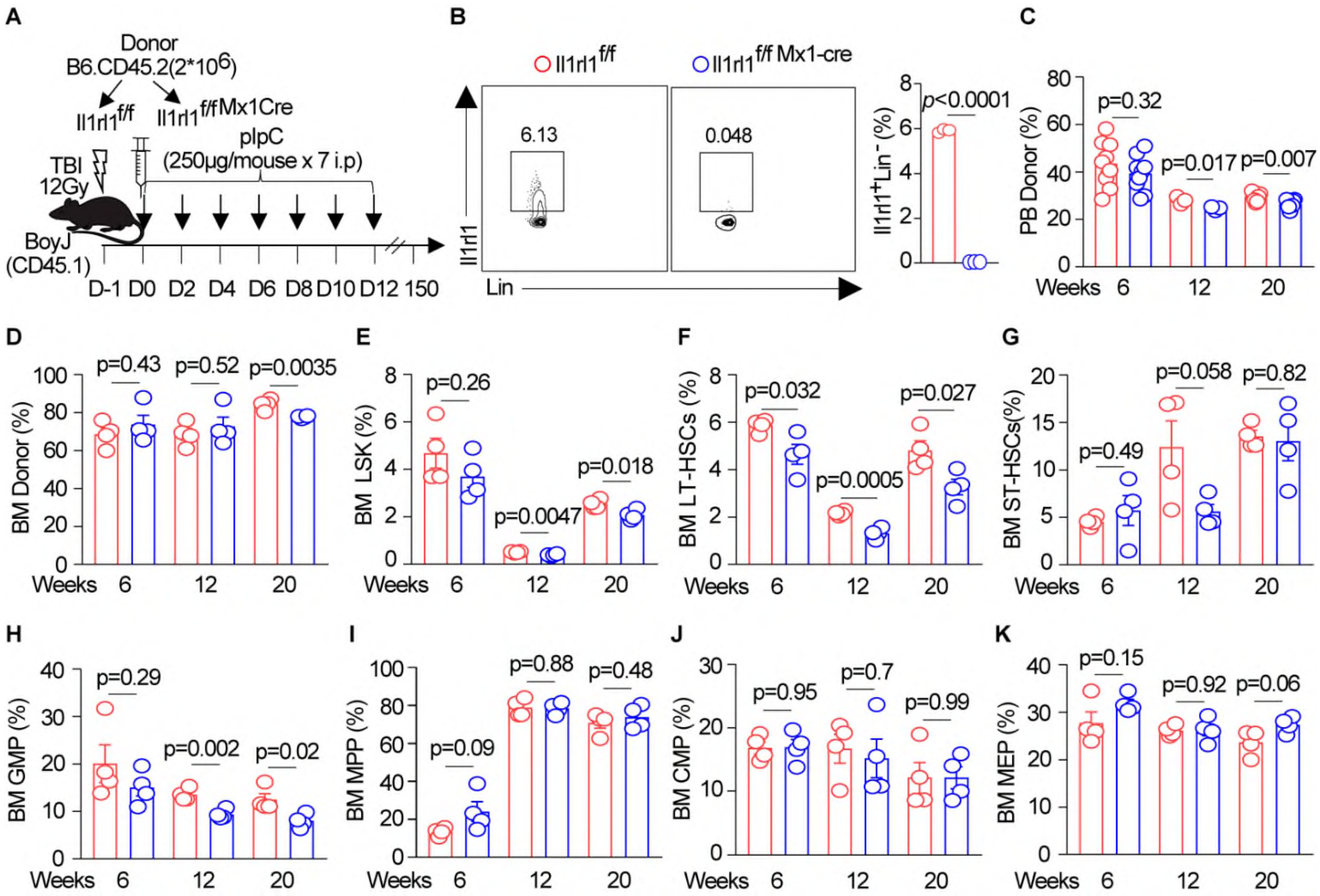

Supplementary Fig. 4. Il1rl1 is indispensable for steady-state hematopoiesis. (**A**) Experimental scheme of Il1rl1 deficiency in normal hematopoiesis. (**B**) Il1rl1 deletion on Lineage negative hematopoietic stem cells post 7 doses of pIpC injection. Frequencies of donor cells in the peripheral blood (**C**), donor cells in the bone marrow (**D**), donor LSK cells, (**E**) donor long-term HSCs (**F**), donor short-term HSCs (**G**), donor granulocyte monocyte progenitors (**H**), multi potent progenitors (**I**), common myeloid progenitors (**J**), megakaryocyte erythrocyte progenitors (**K**) in Il1rl1$^{f/f}$ and Il1rl1$^{f/f\ Mx1Cre}$ recipient mice at 6-, 12, 20 weeks post-transplantation. Data are mean ± s.e.m. (n = 4-9); Unpaired two-sided t-test was used.

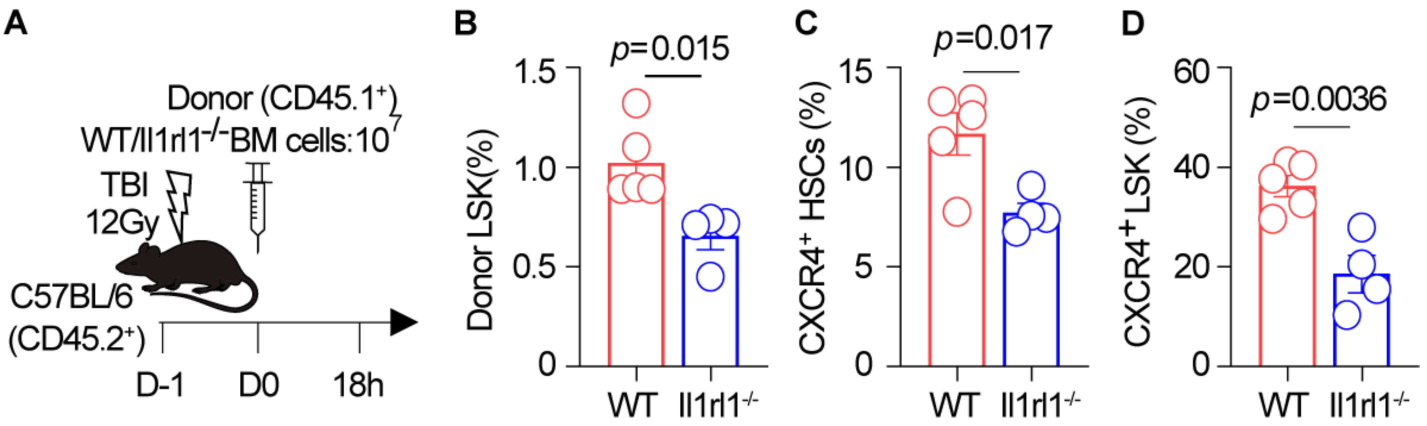

Supplementary Fig. 5. Homing potential of WT and Il1rl1$^{-/-}$ BM cells. (**A**) Experimental scheme. (**B**) Frequency of donor Lin^{-}c-KIT^{+}Sca-1^{+} cells in the recipient mice transferred with WT vs. Il1rl1$^{-/-}$ BM cells. (**C**) Frequency of donor CXCR4^{+}HSCs cells in the recipient mice transferred with WT vs. Il1rl1$^{-/-}$ BM cells. (**D**) Frequency of donor CXCR4^{+}LSK cells in the recipient mice transferred with WT vs. Il1rl1$^{-/-}$ BM cells. n=4/group. Data are mean ± s.e.m. (n =4); Unpaired two-sided t-test was used.

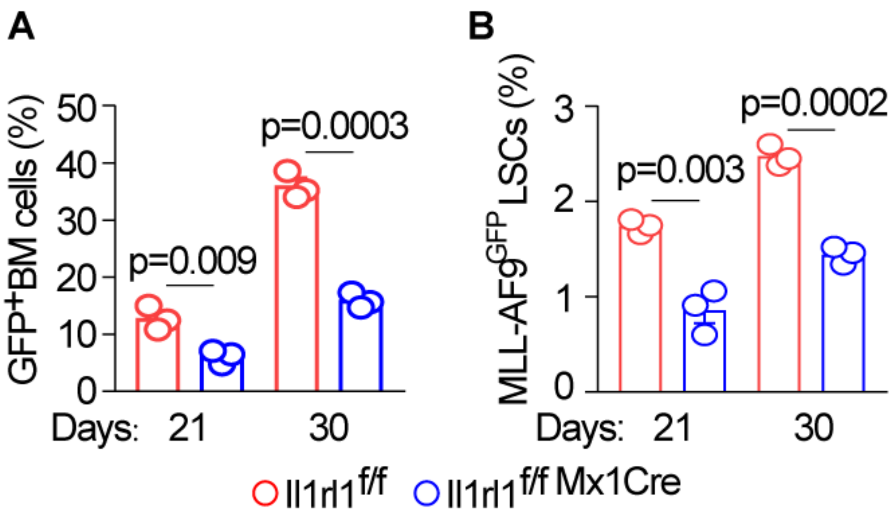

Supplementary Fig. 6. Frequencies of GFP⁺ leukemic cells and MLL-AF9 leukemic stem cells (LSCs) in the BM of mice from the leukemogenesis initiation model. Frequencies of GFP⁺ leukemic cells (**A**) and MLL-AF9^{GFP} leukemic stem cells (**B**) in the BM from Il1rl1$^{f/f}$ and Il1rl1$^{f/f\ Mx1Cre}$ transferred mice at Day 21 and 30**.** Data are mean ± s.e.m. (n=3); Unpaired two-sided t-test was used.

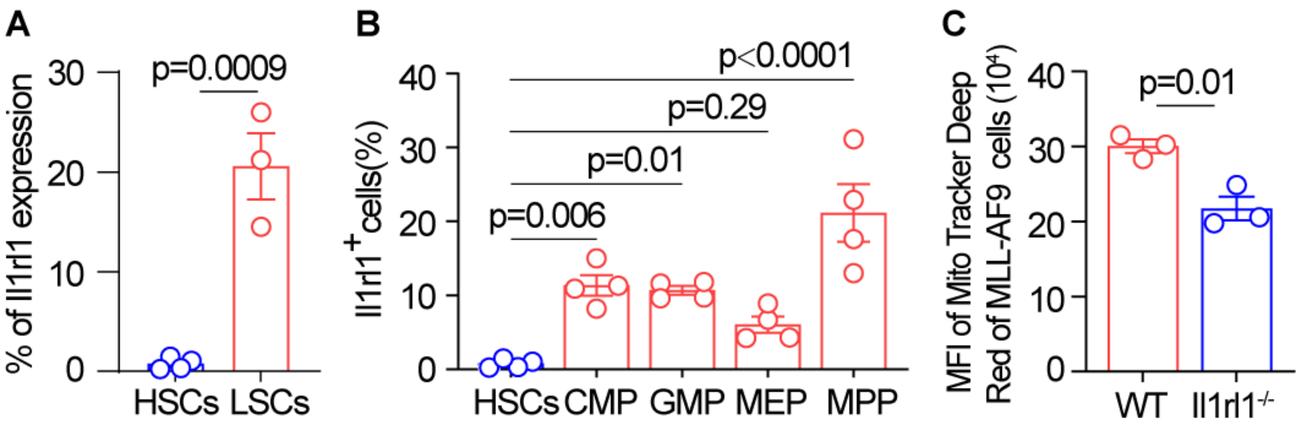

Supplementary Fig. 7. Il1rl1 expression in murine HSCs, LSCs, and LPSCs, and Mito Tracker Deep Red of MLL-AF9 leukemic cells. (**A**) Frequencies of Il1rl1^{+}HSCs at steady state and LSCs at day21 post-challenge. (**B**) Frequencies of Il1rl1^{+}HSCs at steady-state and Il1rl1^{+}CMP, GMP, MEP and MPP from WT leukemic cells transferred mice at day 21 post-challenge. (**C**) MFI of Mito Tracker Deep red of WT or Il1rl1$^{-/-}$ MLL-AF9 leukemic cells at day 21 post-challenge. Data are mean ± s.e.m. (n=3-4). Unpaired two-sided t-test was used for panel A and C. ANOVA test was used for panel B.

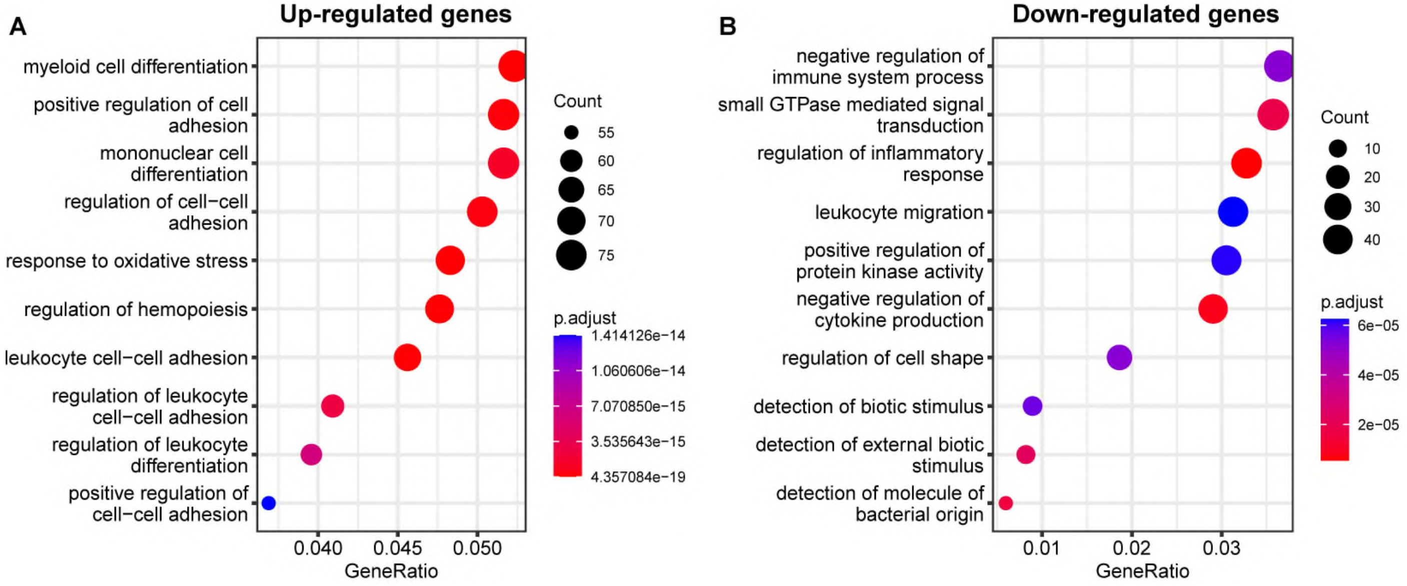

Supplementary Fig. 8. KEGG pathway analysis of differentially expressed genes between WT and Il1rl1$^{-/-}$ MLL-AF9 LSCs. (**A**) KEGG pathway analysis of up-regulated genes. (**B**) KEGG pathway analysis of down-regulated genes.

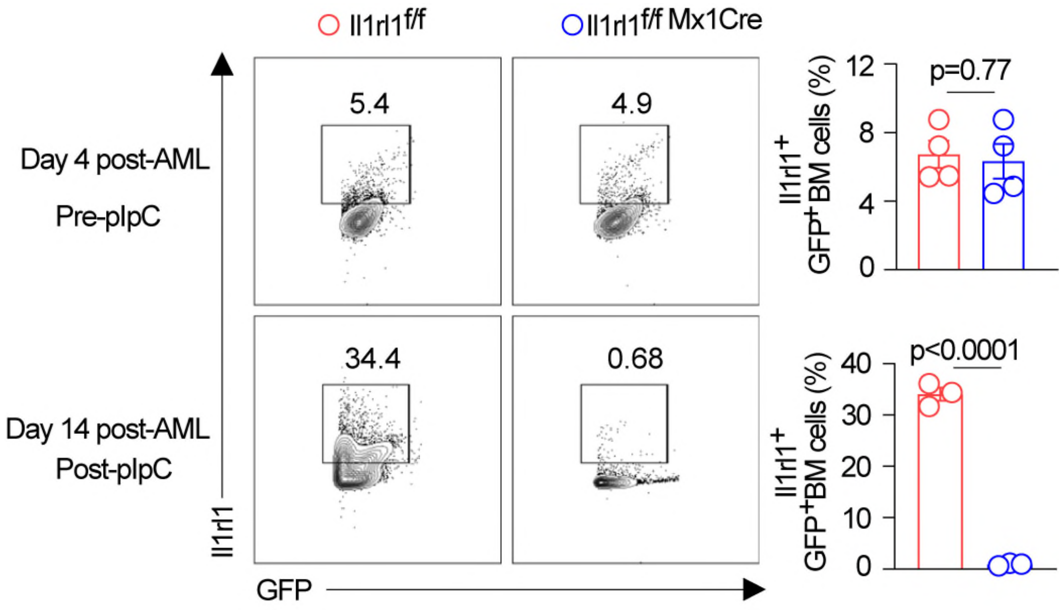

Supplementary Fig. 9. Il1rl1 expression in MLL-AF9 leukemic cells in the BM of mice from the leukemia maintenance model before and after pIpC administration. Representative flow plots of Il1rl1⁺GFP BM cells and statistical analysis of Il1rl1⁺GFP⁺ BM cells in Il1rl1$^{f/f}$ and Il1rl1$^{f/f\ Mx1Cre}$ transferred mice on day 4 before and day 14 after pIpC administration (250μg/kg). Data are mean ± s.e.m. (n=3-4); Unpaired two-sided t-test was used.

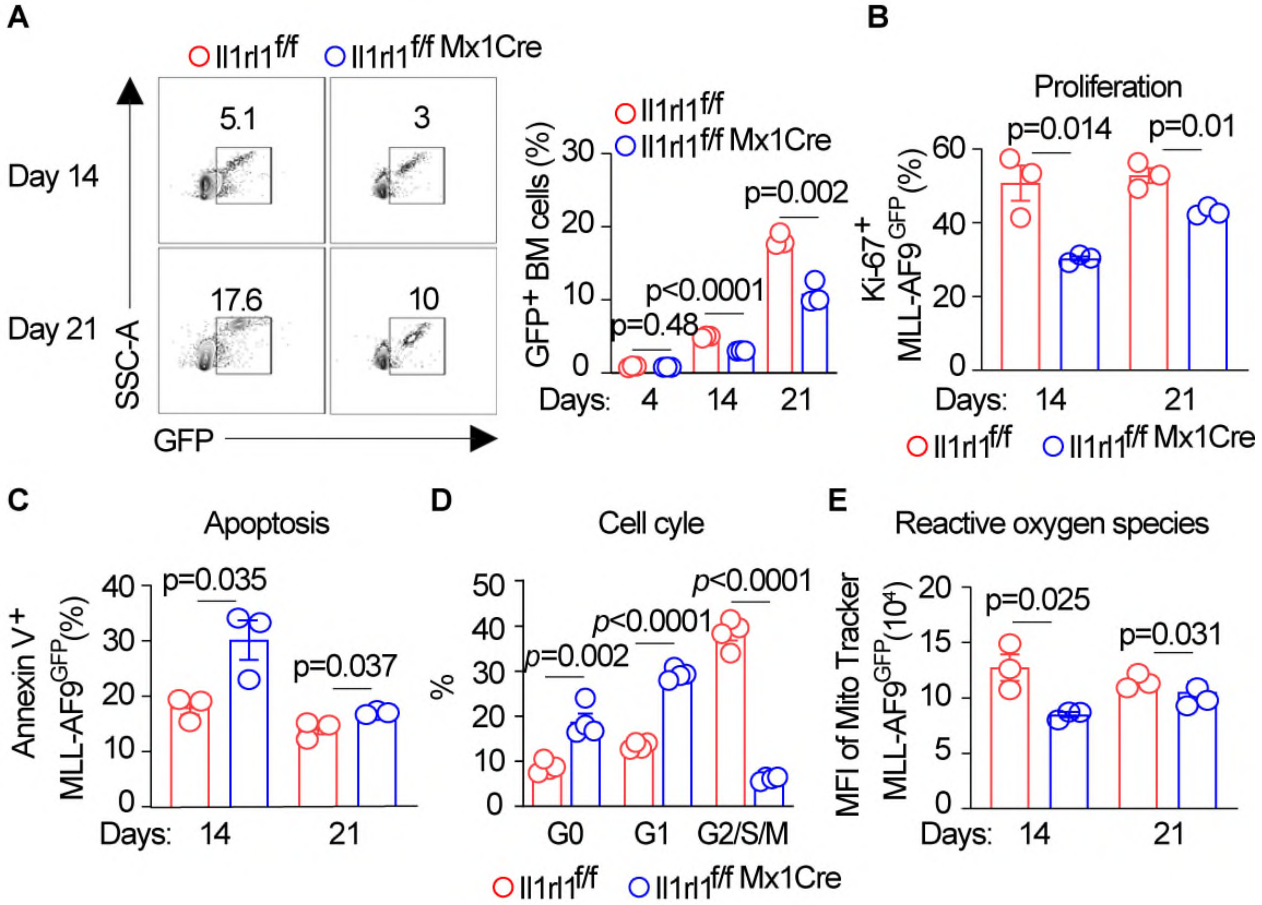

Supplementary Fig. 10. Frequencies, proliferation, apoptosis, and reactive oxygen species of GFP⁺ leukemic cells in the leukemia maintenance model. Representative flow plots and frequencies of GFP⁺ leukemic cells **(A)**, Ki-67⁺MLL-AF9GFP (**B**), Annexin V⁺MLL-AF9GFP (**C**), Cell cycle (**D**), and MFI of Mito Tracker MLL-AF9GFP (**E**) in the malignant BM niches of Il1rl1f/f and Il1rl1f/f Mx1Cre LSCs transferred leukemic mice on Day 14 and Day 21 post-challenge. Data are mean ± s.e.m. (n =3-4); Unpaired two-sided t-test was used.

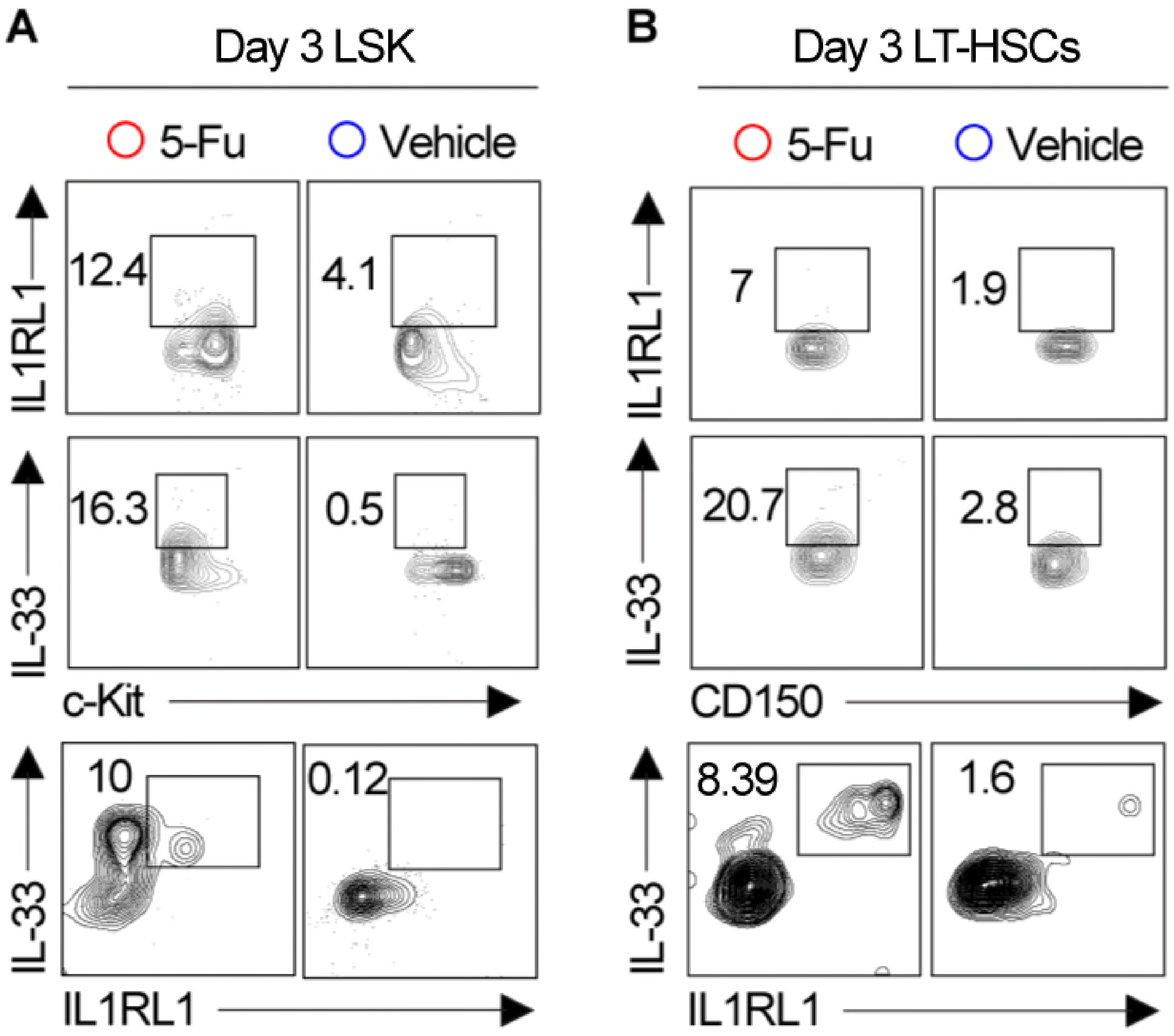

Supplementary Fig. 11. Stress induced IL-33/Il1rl1 signaling autocrine loop in normal hematopoietic stem cells. (A) Representative flow plots of $Il1rl1^+$LSK (Lin^- $c\text{-}KIT^+$ $Sca\text{-}1^+$, LSK), $IL\text{-}33^+$LSK, and $Il1rl1^+IL\text{-}33^+$LSK from the $IL\text{-}33^{GFP}$ reporter mice at day 3 post one dose 5-Fu (150mg/kg) and vehicle administration. **(B)** Representative flow plots of $Il1rl1^+$LT-HSCs (gating on $CD150^+CD48^-Lin^-$ $c\text{-}KIT^+$ $Sca\text{-}1^+$), $IL\text{-}33^+$LT-HSCs, and $Il1rl1^+IL\text{-}33^+$LT-HSCs from the $IL\text{-}33^{GFP}$ reporter mice at day 3 post one dose 5-Fu (150mg/kg) and vehicle administration.

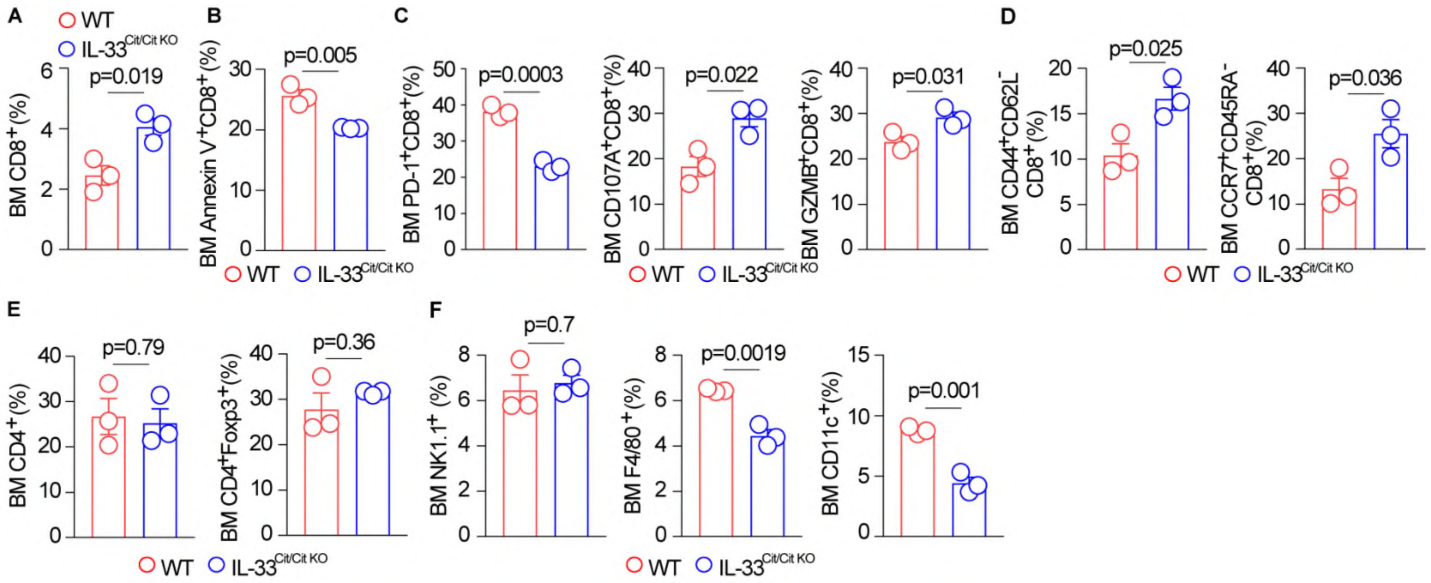

Supplementary Fig. 12. Leukemia immune microenvironment cell subsets analysis of IL-33/Il1rl1 signaling autocrine loop model. (**A**) Frequency of $CD3^+CD8^+$T cells, (**B**) Annexin V^+CD8^+ T cells, (**C**) PD-1^+, $CD107A^+$, $GZMB^+CD8^+$T cells, (**D**) $CD44^+CD62L^-CD8^+$ and $CCR7^+CD45RA^-CD8^+$T cells, (**E**) $CD4^+$, $CD4^+Foxp3^+$T cells and (**F**) NK1.1^+, F4/80^+, $CD11c^+$ cells in the malignant BM niches from WT vs. IL-$33^{Cit/Cit\ KO}$ LSCs transferred mice on Day 14. Data are mean ± s.e.m. (n =3); Unpaired two-sided t-test was used.

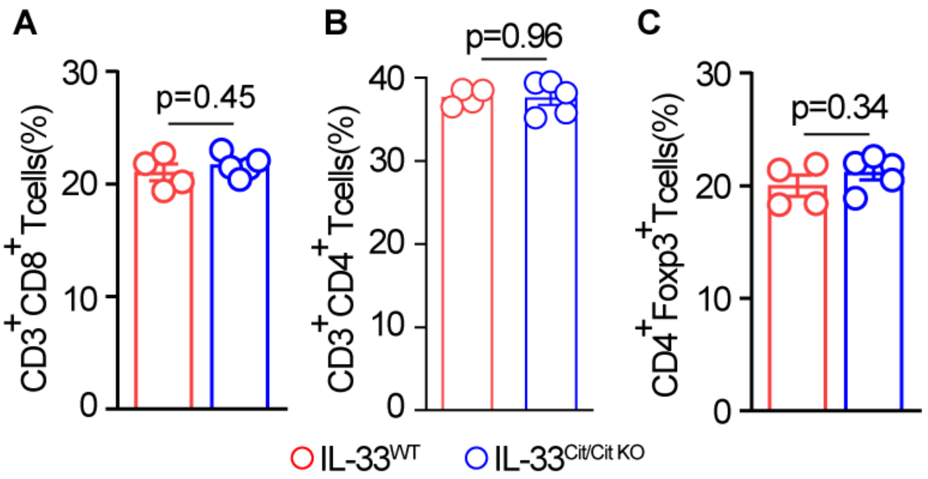

Supplementary Fig. 13. Frequencies of CD8$^+$, CD4$^+$, and regulatory T cells in non-leukemic wild type and IL-33$^{cit/cit\ KO}$ mice at steady state. (**A**) CD3$^+$CD8$^+$T cells; (**B**) CD3$^+$CD4$^+$T cells (**C**) CD4$^+$Foxp3$^+$T cells. Data are mean ± s.e.m. (n =4-5); Unpaired two-sided t-test was used.

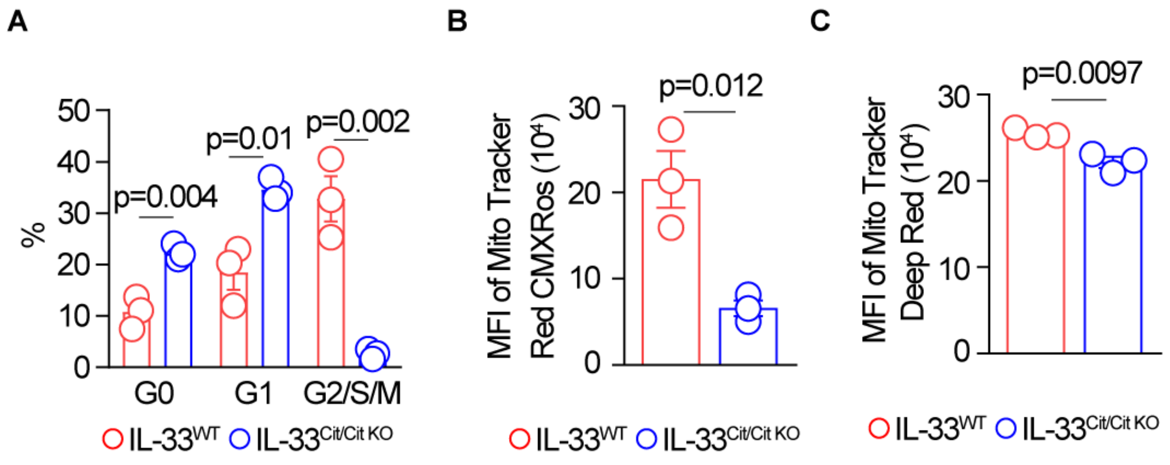

Supplementary Fig. 14. Cell cycle and reactive oxygen species of GFP^{+} leukemic cells in the IL-33/Il1rl1 signaling autocrine loop leukemia model. Cell cycle Ki-67^{+}MLL-AF9^{GFP}**(A)**, and MFI of Mito Tracker Red CMXRos **(B)** and Deep Red (**C**) of MLL-AF9^{GFP} cells in the malignant BM niches of IL-33^{WT} and IL-33$^{cit/cit\ KO}$ LSCs transferred leukemic mice on Day 14 post-challenge. Data are mean ± s.e.m. (n =3); Unpaired two-sided t-test was used.

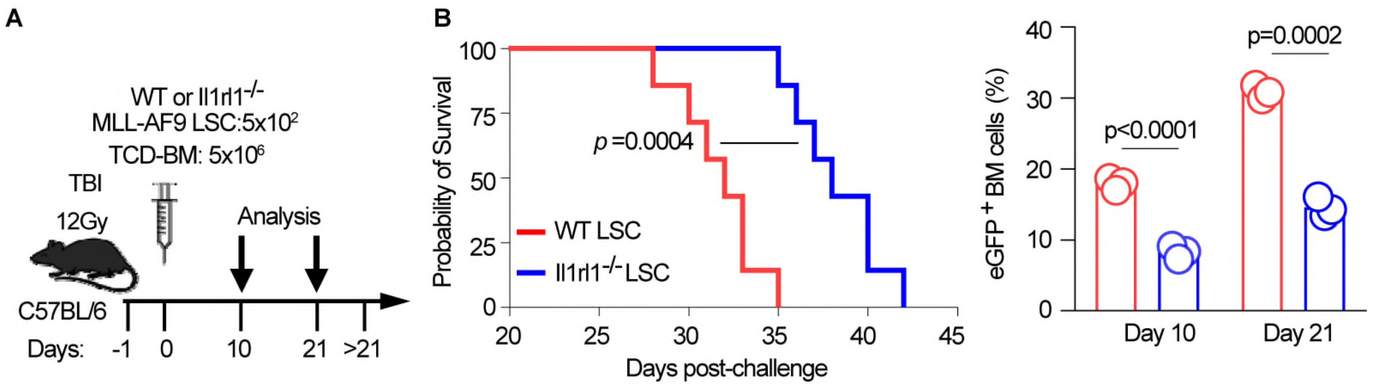

Supplementary Fig. 15. Il1rl1 deficiency in MLL-AF9 LSC inhibits their growth. (A) Schema of Il1rl1 deficiency in LSCs in MLL-AF9 model. (B) Kaplan-Meier curve and leukemia burden of mice transferred with WT and Il1rl1-deficient LSCs. Data are mean ± s.e.m. (n =3); Unpaired two-sided t-test was used.

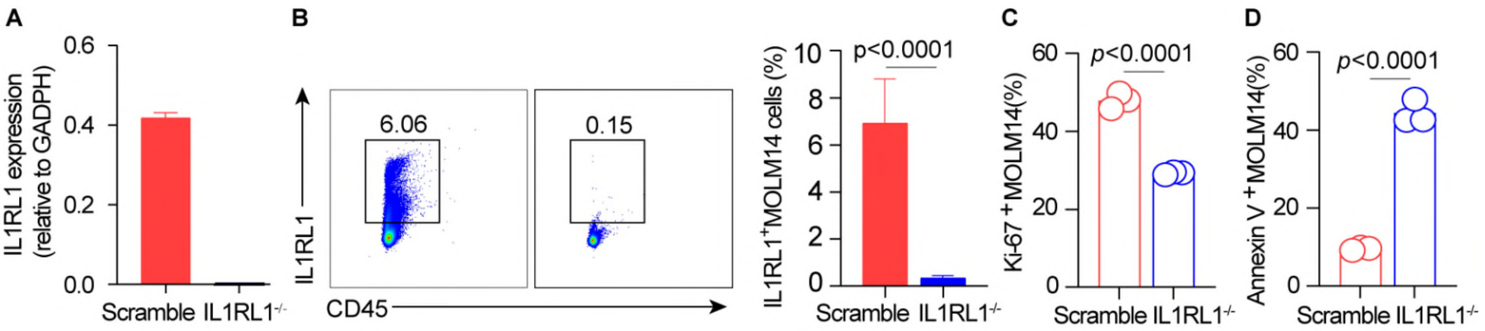

Supplementary Fig. 16. **IL1RL1 deficiency in MOLM14 cells decreases their proliferation and increases apoptosis**. IL1RL1 deletion in MOLM14 cells verified using qPCR (**A**), and IL1RL1^{+}MOLM14 cells by flow cytometry staining (**B**). Percentages of Ki-67^{+} (**C**), and Annexin V^{+} (**D**) in IL1RL1$^{-/-}$ vs Scramble control MOLM14 cells. Data are mean ± s.e.m. (n = 4); Unpaired two-sided t-test was used for C-D.

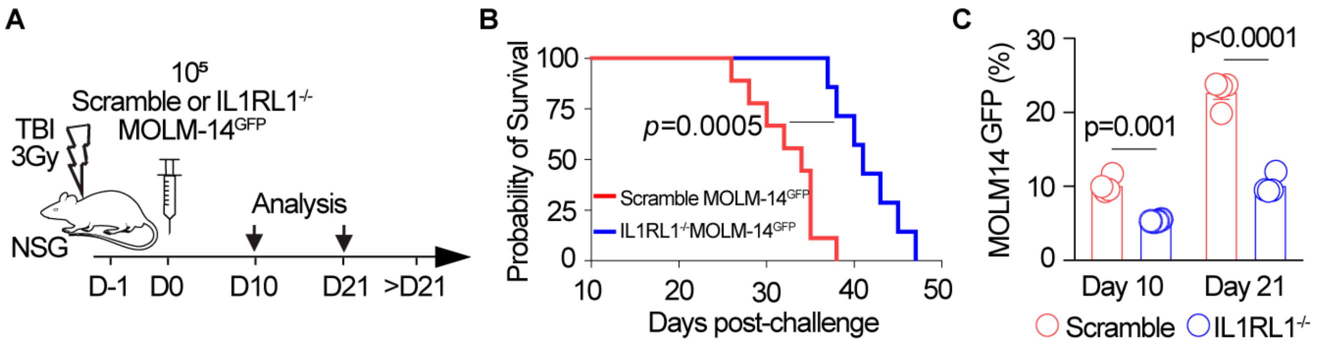

Supplementary Fig. 17. IL1RL1 deficiency in MOLM14 cells inhibits their growth to extend survival. (A) Experimental design for investigating the role of IL1RL1 in a human leukemia model. **(B)** Kaplan-Meier survival curve of mice transplanted with scramble or IL1RL1$^{-/-}$ MOLM14^{GFP} cells. **(C)** Frequencies of MOLM14^{GFP} leukemia cells in the bone marrow of mice transplanted with Scramble or IL1RL1$^{-/-}$ MOLM14 cells on Day 10 and 21**.** Data are mean ± s.e.m. (n = 4); Unpaired two-sided t-test was used for C.

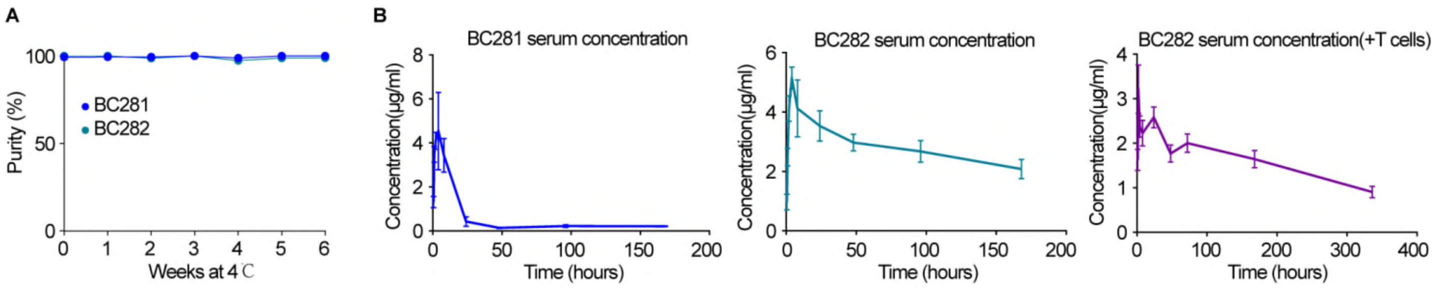

Anti-IL1RL1 BsAb	Half life	Half life	Tmax	Cmax	Clast	AUCall	Vz_obs	Cl_obs
	hr	days	hr	ug/mL	ug/mL	hr*ug/mL	mL	mL/hr
mIl1rl1(BC281)	8.51	0.35	4	4.54	0.21	92.16	1.2961	0.1056
hIL1RL1 (BC282)	186.75	7.78	4	5.14	2.08	488.65	2.5682	0.0095
BC282+T cells	244.60	10.19	2	3.21	0.9	539.94	4.1151	0.0115

Supplementary Fig. 18. Stability and pharmacodynamic study of anti-IL1RL1 T-BsAbs. (**A**) Stability analysis of anti-IL1RL1 T-BsAbs at 4℃ for over 6 weeks. (**B**) Pharmacodynamic study of BC281 and BC282 *in vivo* employing C57BL/6 or NSG background mice (n = 5). The provided table shows half-lives, time point with the highest concentration (Tmax), maximum concentration (Cmax), concentration at the last timepoint (Clast) and area under the curve for the study (AUCall, hr*μg/mL). Data are mean ± s.e.m.

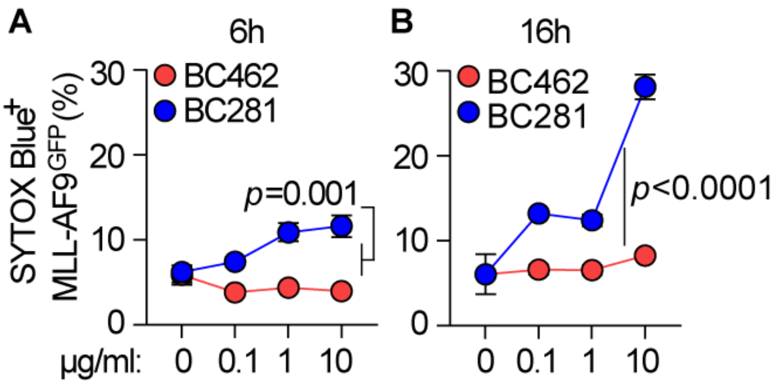

Supplementary Fig. 19. Cytotoxicity of anti-mIl1rl1 T-BsAbs on MLL-AF9 cells. Cytotoxicity of anti-mIl1rl1 T-BsAbs at different concentrations on MLL-AF9 cells at 6 **(A)** and 16 **(B)** hours (E:T=10:1). Two-way ANOVA was used.

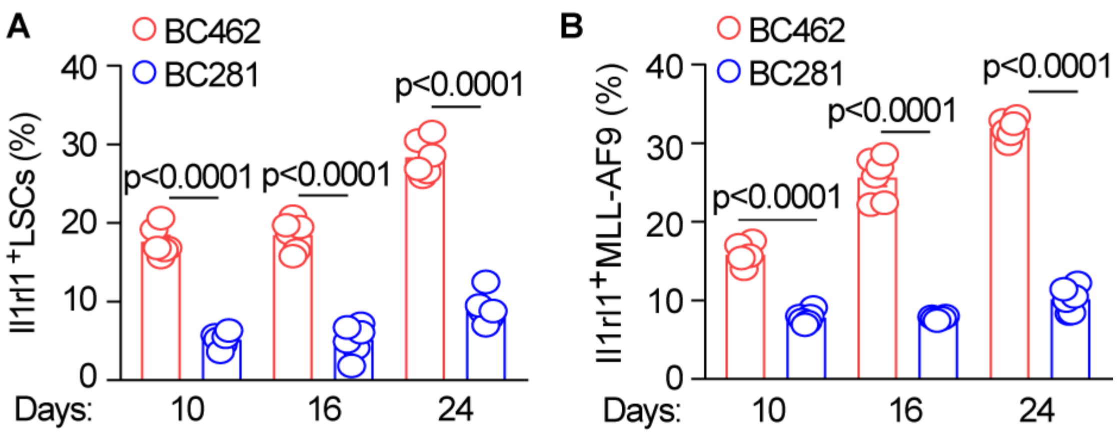

Supplementary Fig. 20. Il1rl1 expression in leukemic cells and LSCs after anti-Il1rl1 T-BsAbs treatment. Frequencies of Il1rl1^{+}LSCs (**A**) and Il1rl1^{+}leukemic cells (**B**) in the BM of mice treated with anti-Il1rl1 T-BsAbs or control T-BsAbs at Day 10, 16, and 24. Data are mean ± s.e.m. (n = 6);Unpaired two-sided t-test was used.

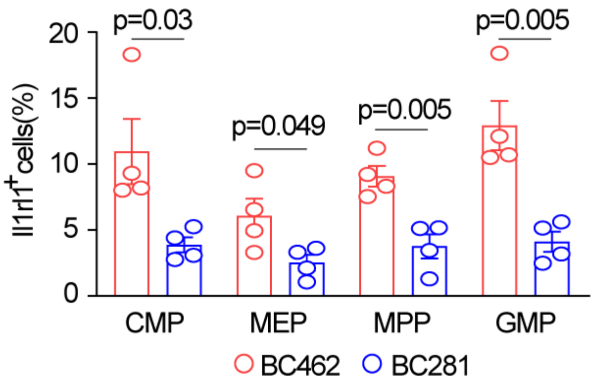

Supplementary Fig. 21. Il1rl1 expression in non-LSC progenitors after anti-Il1rl1 T-BsAbs treatment at Day 10. CMP (common myeloid progenitor), MEP (megakaryocyte-erythrocyte progenitor), MPP (multipotent progenitor), and GMP (granulocyte-monocyte progenitor). Data are mean ± s.e.m. (n = 4); Unpaired two-sided t-test was used.

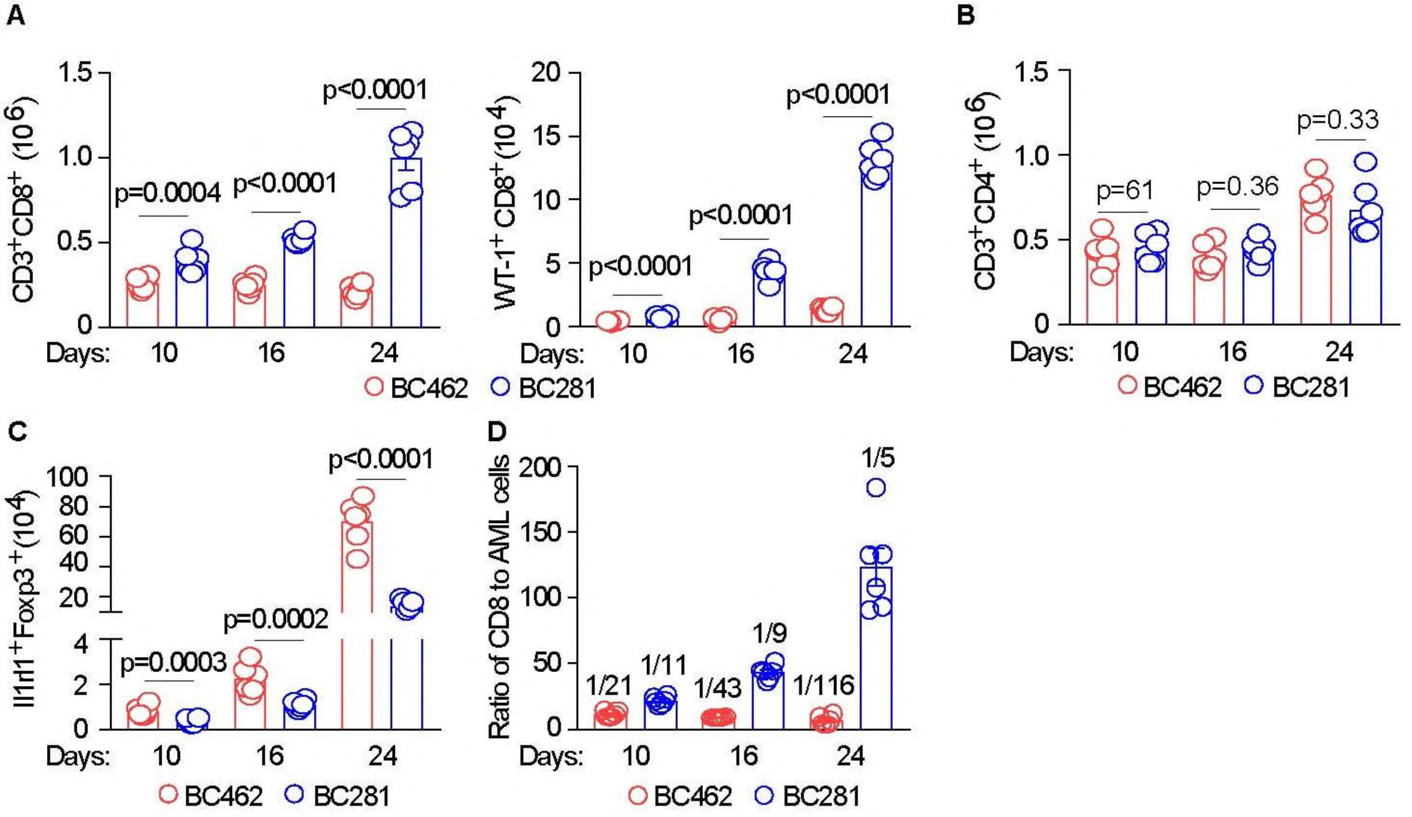

Supplementary Fig. 22. Absolute numbers of CD3⁺CD8⁺, WT-1⁺CD8⁺, CD3⁺CD4⁺, and Il1rl1⁺Foxp3⁺T cells, and the CD8⁺T cells to leukemic cells ratio in BC281- vs. BC462-treated leukemic mice. Absolute number of CD3⁺CD8⁺ and WT-1⁺CD8⁺(**A**), CD3⁺CD4⁺(**B**), Il1rl1⁺Foxp3⁺ T regs (**C**), and ratio of CD8 T cells to leukemic cells (**D**) in the BM of BC281 vs BC462 treated leukemic mice at Day 10, 16, and 24 post-treatment. Data are mean ± s.e.m. (n = 6). Unpaired two-sided t-test was used.

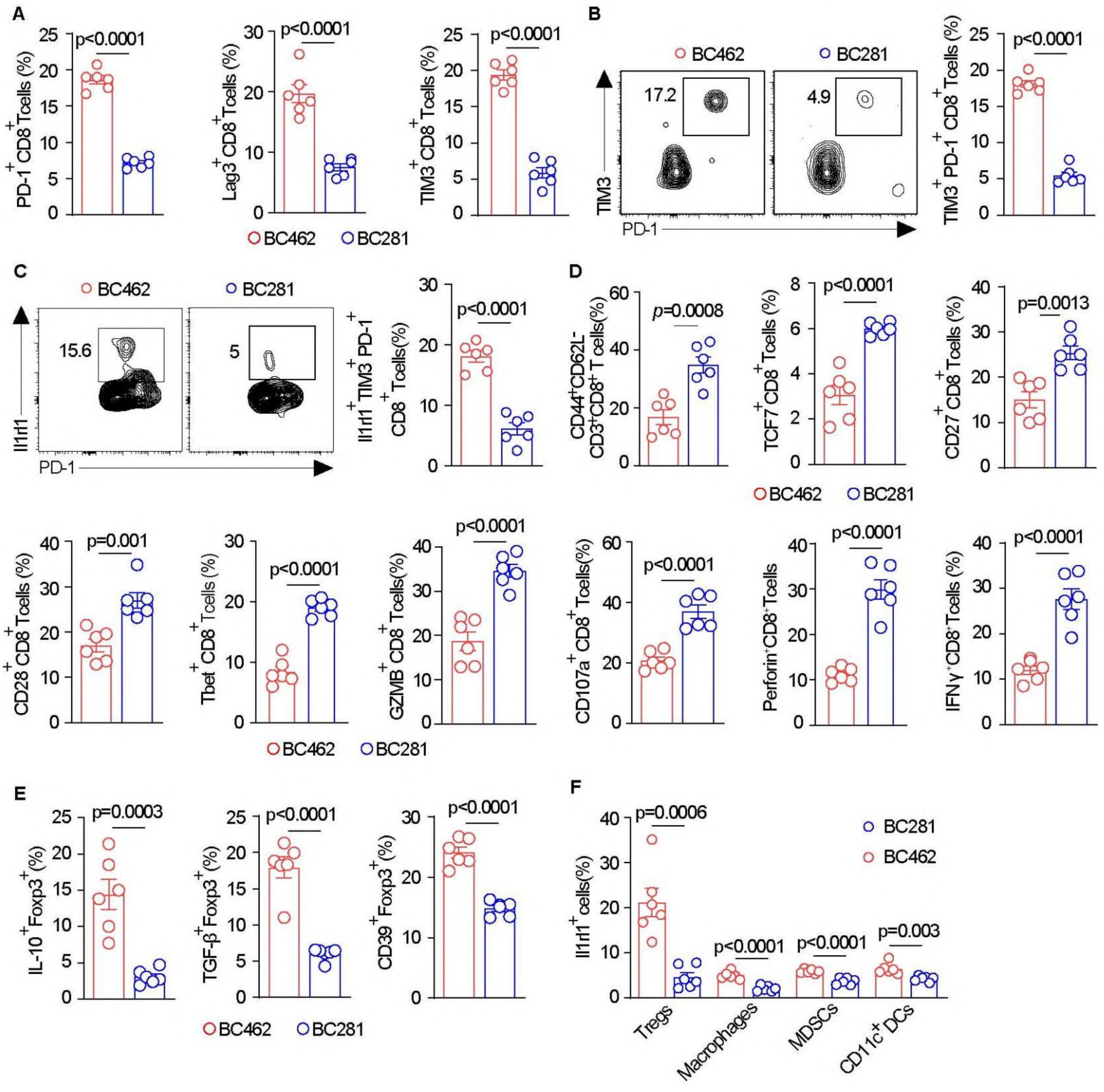

Supplementary Fig. 23. Anti-Il1rl1 T-BsAb reverses the immunosuppressive microenvironment. (A) Frequencies of PD-1^{+}CD8^{+}, Lag3^{+}CD8^{+}, TIM3^{+}CD8^{+} T cells in the BM of leukemic mice on day 10 post BC281 and control BC462 treatment. (**B**) Representative flow plots and frequencies of PD-1^{+}TIM3^{+}CD8^{+} T cells in the BM of leukemic mice on day 10 post BC281 and control BC462 treatment. (**C**) Presentative flow plots and frequencies of Il1rl1^{+}PD-1^{+}TIM3^{+}CD8^{+} T cells in the BM of leukemic mice on day 10 post BC281 and control BC462 treatment. (**D**) CD44+CD62L^{-}CD8^{+}, TCF7^{+}CD8^{+}, CD27^{+}CD8^{+}, CD28^{+}CD8^{+}, Tbet^{+}CD8^{+}, GZMB^{+}CD8^{+},

CD107a$^+$CD8$^+$, Perforin$^+$CD8$^+$, IFN-γ$^+$CD8$^+$ T cells in the BM of BC281 and control BC462 treated leukemic mice. **(E)** IL-10$^+$Foxp3$^+$, TGF-b$^+$Foxp3$^+$, and CD39$^+$Foxp3$^+$ T cells in the BM of BC281 and control BC462 treated leukemic mice. **(F)** Il1rl1 expression in Tregs, macrophages, MDSCs, and CD11c$^+$DCs in the BM of BC281 and control BC462 treated leukemic mice. Data are mean ± s.e.m. (n = 3-6); Unpaired two-sided t-test was used.

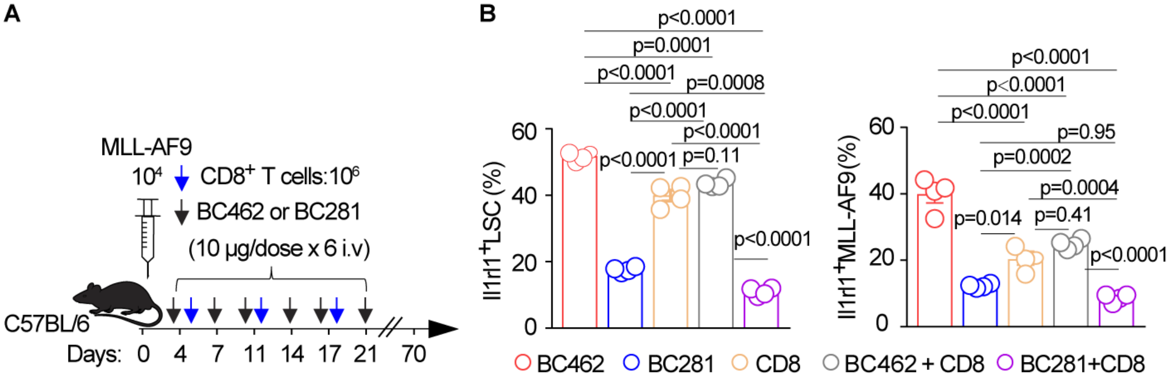

Supplementary Fig. 24. Il1rl1 expression in leukemic cells and LSCs following anti-Il1rl1 T-BsAb treatment in combination with adoptive transfer of CD8⁺T cells. (**A**) Experimental schema. (**B**) Frequencies of Il1rl1⁺LSCs and Il1rl1⁺leukemic cells in the BM of leukemic mice treated with BC281, BC462, CD8⁺T cells alone, or in combination with adoptive transfer of CD8⁺T cells at Day 28 post-treatment. Data are mean ± s.e.m. (n = 6); ANOVA was used for B.

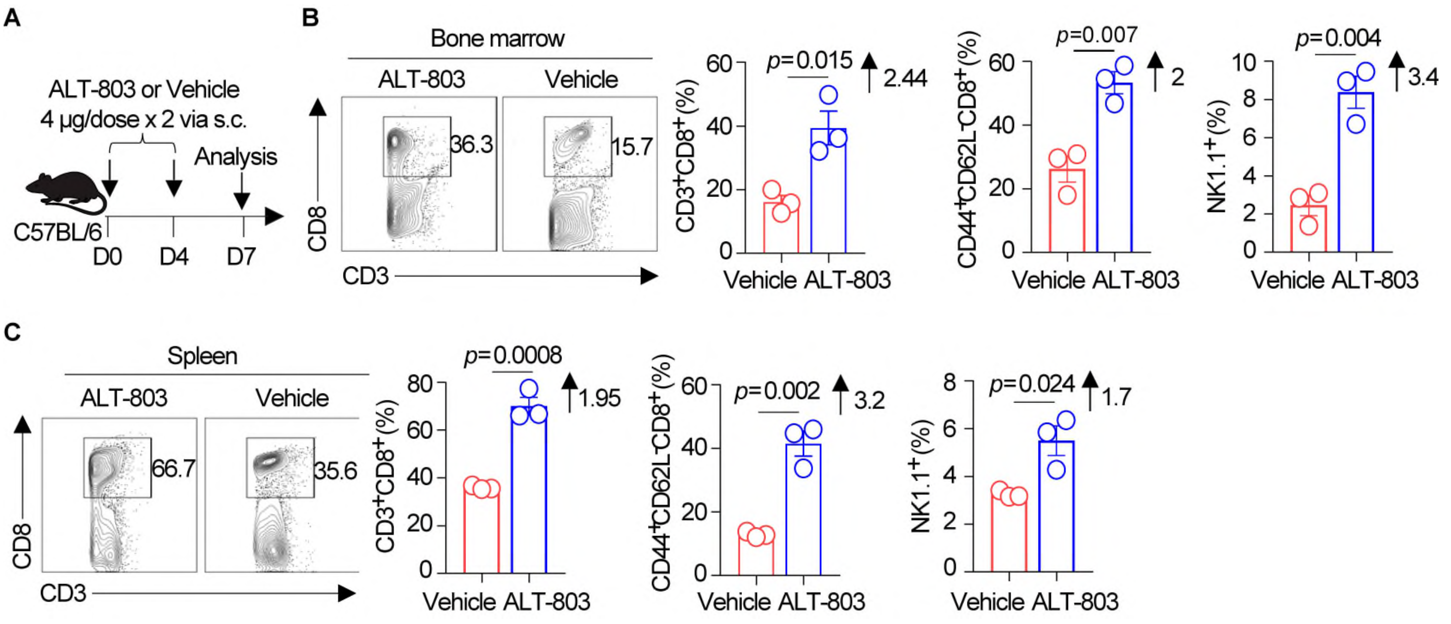

Supplementary Fig. 25. Functional test of ALT-803 *in vivo*. (A) Experimental scheme. (**B**) Representative flow plots and frequencies of $CD3^{+}CD8^{+}$T cells, $CD44^{+}CD62L^{-}CD8^{+}$T cells, $NK1.1^{+}$ cells in the bone marrow of mice treated with ALT-803 vs vehicle on day 7 post-injection. **(C)** Representative flow plots and frequencies of $CD3^{+}CD8^{+}$T cells, frequencies of $CD44^{+}CD62L^{-}CD8^{+}$T cells, $NK1.1^{+}$ cells in the spleen of mice treated with ALT-803 vs vehicle on day 7 post-injection. Data are mean ± s.e.m. (n = 3); Unpaired two-sided t-test was used.

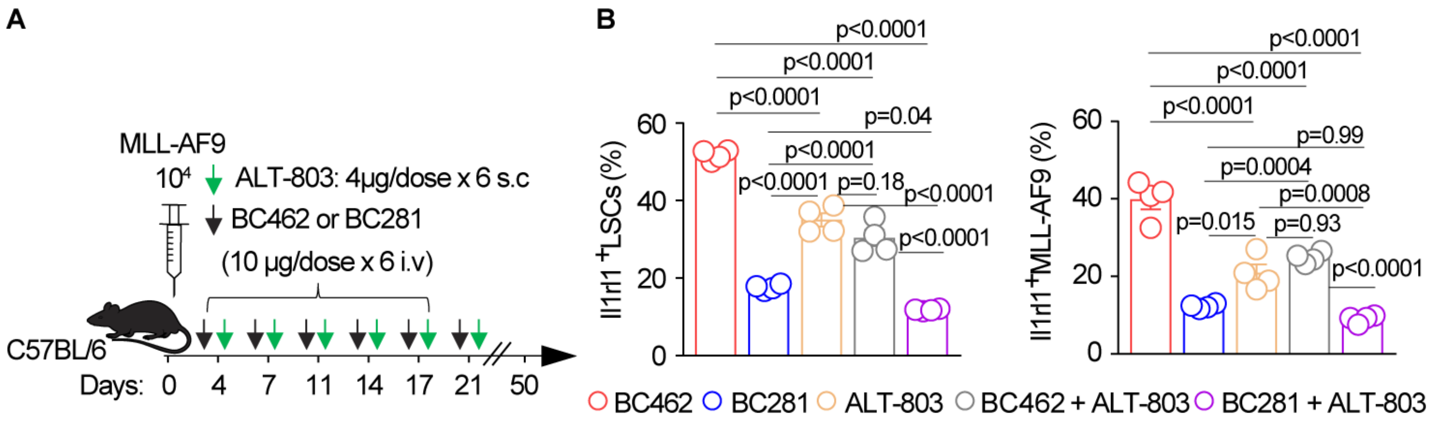

Supplementary Fig. 26. Il1rl1 expression in leukemic cells and LSCs following anti-Il1rl1 T-BsAb treatment in combination with ALT-803 model. (**A**) Experimental schema of BC281 treatment in combination with ALT-803. (**B**) Frequencies of Il1rl1$^+$LSCs and Il1rl1$^+$MLL-AF9 in the BM of mice treated with BC281, BC462, ALT-803 alone, BC281 plus ALT-803, and BC462 plus ALT-803 at Day 30. Data are mean ± s.e.m. (n = 4/group); ANOVA was used for B.

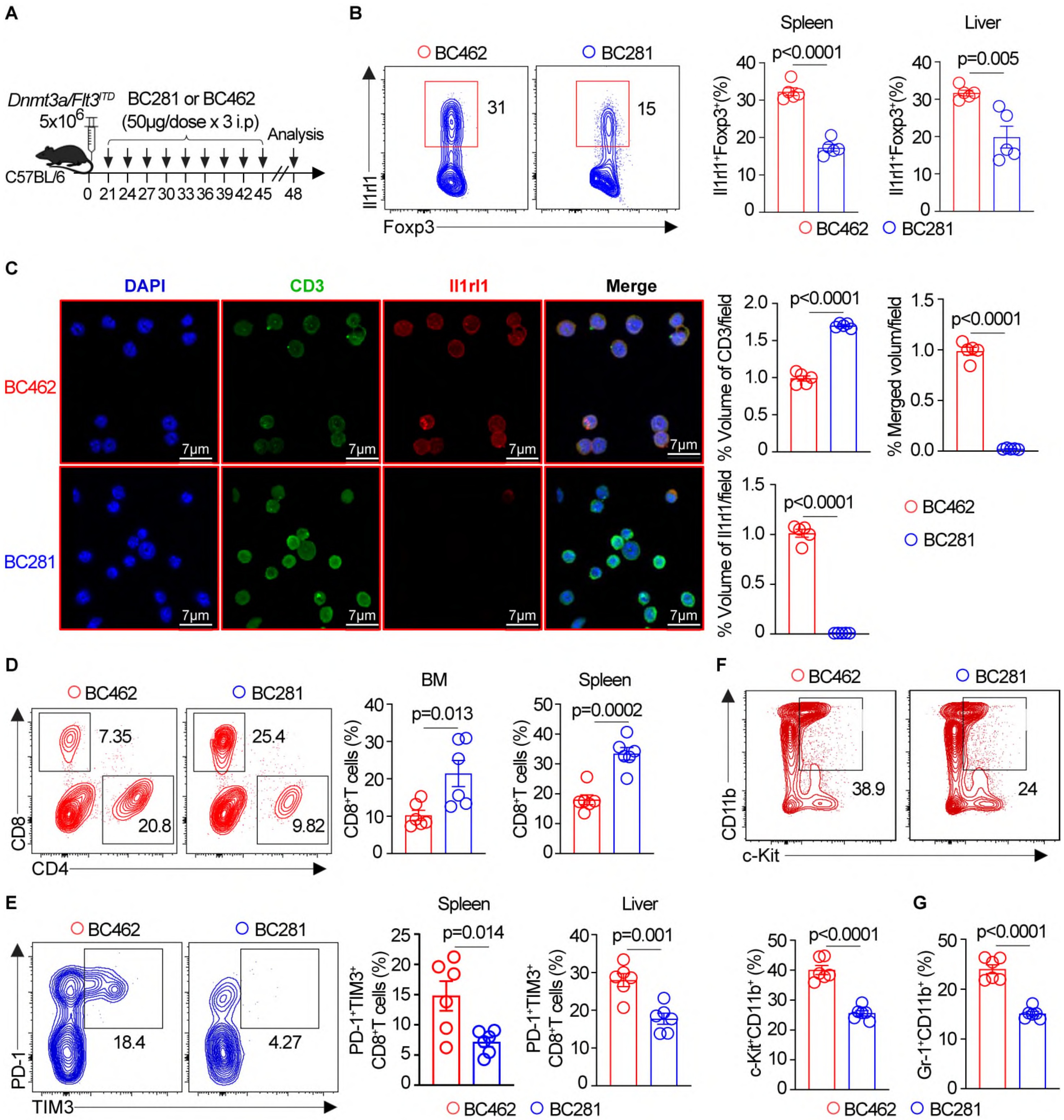

Supplementary Fig. 27. Dual targeting with anti-Il1rl1 T-BsAb in an epigenetically induced immunocompetent myeloid leukemia model.

(**A**) Anti-murine Il1rl1 T-BsAb treatment schema in the DNMT3A/FLT3^{ITD} immunocompetent myeloid leukemia model. (**B**) Representative plots of Il1rl1^{+}Foxp3^{+}T cells gating in the spleen from the leukemic mice and

frequencies of Il1rl1^{+}Foxp3^{+}T cells in the spleen and liver in the BC281 vs BC462 treated leukemic mice (n=6/group). (**C**) Representative confocal images showing DAPI (blue), anti-CD3 (green), anti-Il1rl1 (red), and merged immunofluorescence staining in the spleens of DNMT3A/FLT3^{ITD} AML mice treated with either BC281 or control BC462. In the spleens of BC281-treated AML mice, Il1rl1^{+}cells are nearly absent, while the number of CD3^{+} T cells is significantly increased compared to the BC462-treated group. (**D**) Representative plot of CD8^{+}T cells gating in the BM from the leukemic mice and frequencies of CD3^{+}CD8^{+}T cells in the BM and spleen in the BC281 vs BC462 treated leukemic mice (n=6/group). (**E**) Representative plots of PD-1^{+}TIM3^{+}CD8^{+}T cells gating in the BM from the leukemic mice and frequencies of PD-1^{+}TIM3^{+}CD8^{+}T cells in the BM and spleen in the BC281 vs BC462 treated leukemic mice (n=6/group). (**F**) Representative plot and frequencies of AML blasts (c-Kit^{+}CD11b^{+}) in the spleen of leukemic mice treated with BC281 and BC462 on Day 30 (n=6/group). (**G**) Frequencies of myeloid-derived suppressor cells (Gr-1^{+}CD11b^{+}) in the BM of leukemic mice treated with BC281 and BC462 on Day 30 (n=6/group). Unpaired two-sided t-test was used.

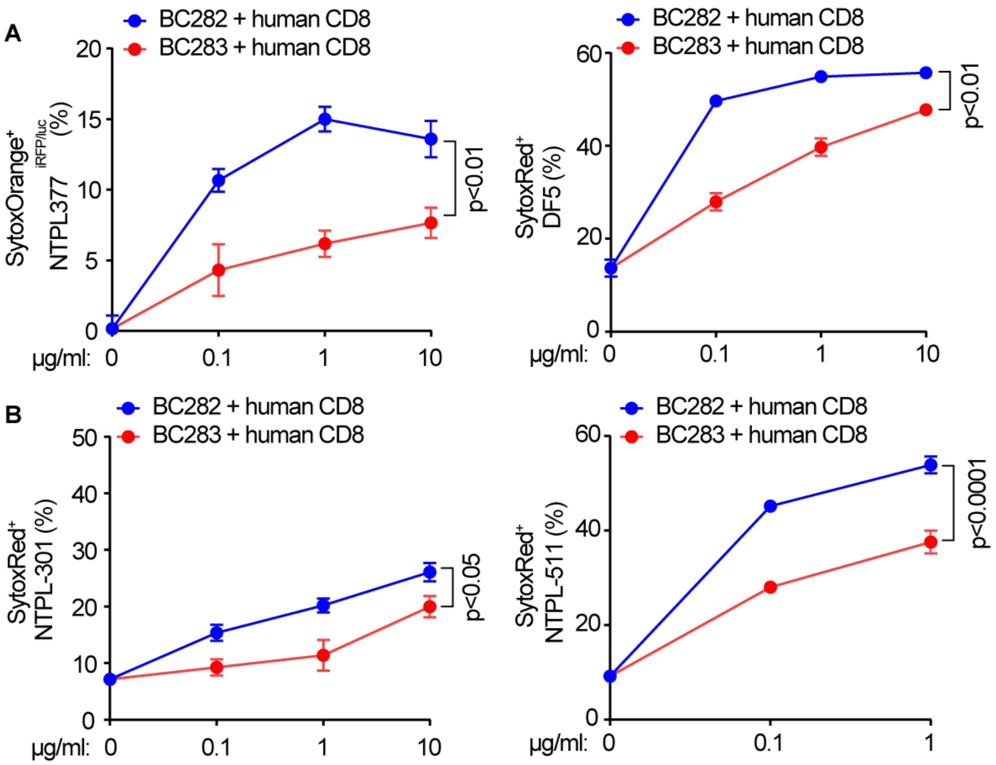

Supplementary Fig. 28. ***In vitro*** **cytotoxicity of anti-IL1RL1 T-BsAbs co-cultured with human $CD8^+$ T cells on pediatric-AML PDX cells.**

(**A**)Two MLL oncofused pediatric-AML PDX cells (NTPL-377, DF-5) stained with SYTOX Orange or Red dye at 16 hours co-culture with human CD8+T cells in the presence of different concentrations of BC282 and control BC283. ANOVA test was used. Two MLL oncofused pediatric-AML PDX cells (NTPL-377, DF-5) stained with SYTOX Orange or Red dye at 16 hours co-culture with human $CD8^+$T cells in the presence of different concentrations of BC282 and control BC283. ANOVA test was used.

(**B**) Two non-MLL oncofused pediatric-AML PDX cells (NTPL-301, NTPL-511) stained with SYTOX Red dye at 16 hours of co-culture with human $CD8^+$T cells in the presence of different concentrations of BC282 and control BC283. ANOVA test was used.

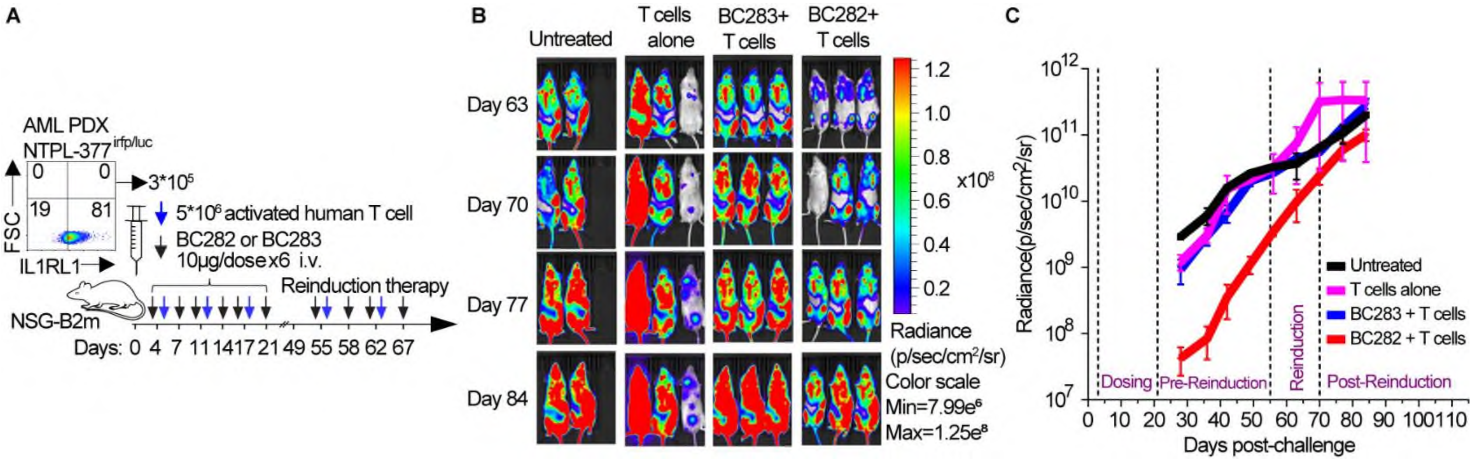

Supplementary Fig. 29. Anti-human IL1RL1 T-BsAb in PDX AML reinduction model. (A) IL1RL1 expression on patients derived leukemic cells NTPL-377, and experimental scheme of anti-hIL1RL1 T-BsAb (BC282) treatment in PDX AML model. **(B)** The luciferase radiance imaging in mice from untreated, T cells treated alone, control BC283 treatment in combination with adoptive transfer of human T cells, and BC282 treatment in combination with adoptive transfer of human T cells on day 63, 70, 77, and 84 post-challenge. **(C)** The quantified luciferase radiance in mice from untreated, T cells treated alone, control BC283 treatment in combination with adoptive transfer of human T cells, and BC282 treatment in combination with adoptive transfer of human T cells on day 28, 36, 42, 49, 63, 70, 77, and 84 post-challenge. Data are mean ± s.e.m. (n =2-5).

Supplementary Table 1. AML patients' demographics

Age	Gender	Race/Ethnicity	Cyto ISCN	FLT3	NPM1	Treatment	% blasts post-induction	Response	% IL1RL1 +LSCs	% IL1RL1+IL-33+LSCs
60	Female	Caucasian	46,XX[20]	Pos	Pos	Tosedostat + DAC	15.50	NR	20.1	5.88
46	Male	Caucasian	43,XY,del(1)(q32),der(1)del(1)(p36.1)t(1;7)(q21;q11.2)add(7)(q36), der(2)inv(2)(p23q35)add(2)(p11.2),del(3)(q21q2?1),del(4)(q22q25),add(5)(q31),-7, der(7)t(1;7),add(8)(p11.2),-11,-12,-13,add(14)(p11.2),add(15)(p11.2), del(15)(q?11.2q24),-16,del(17)(p11.2),add(20)(p13),add(21)(q22),+2~4mar[cp6]/ 41~43,sl,-18[cp3]/41~43,sl,del(18)(p11.1p11.2)[cp3]//46,XX[8]	Neg	Neg	IAP	24.00	NR	18.8	5.52
40	Female	Caucasian	46,XX[20]	Pos	Pos	7 + 3 + Sorafenib	0.50	NR	23.2	19.5
57	Female	Caucasian	ND	ND	ND	FLAM	0.00	CR	9	4.55
56	Female	American Indian or Alaska Native	46XX[20]	ND	ND	IAP	2.00	NR	12	7.14
26	Male	Caucasian	46,XY[20]	Neg	Neg	7 + 3 + GO	0.00	CR	10.1	4.2
68	Male	Unknown	46, XY[20]	Neg	Neg	7 + 3 (DNR90)	0.00	CR	3	1.24
24	Female	Hispanic	46,XX,inv(16)(p13q22)(17]/47,sdl,+22[3]	Neg	Neg	7 + 3 (DNR90)	0.00	CR	7.78	2.99
63	Female	Caucasian	46,XX[20]	Neg	ND	Benda + Idarubicin	5.00	NR	18.8	5
29	Female	Caucasian	46,XX,[20]	Neg	Neg	GCLAC	0.00	CR	10.1	4.2
69	Female	Caucasian	45,X,-X,del(20)(q12)[4]/43,sl,add(3)(q11.2),der(4)t(3;4)(q21;q21),-5,-7,-15,-16,+r,+mar1[7]/44,sdl,+mar2[3]/38,sl,add(3)(q11.2),der(4)t(3;4)(q21;q21),-5,-7,-12,dic(15;21)(p13;p13),-16,-18,-21,add(22)(p11.2)[4]	Neg	Neg	Benda + Idarubicin	24	NR	12.4	12.6
64	Male	Pacific Islander	48,XY,add(3)(q11.2),add(5)(q22),+10,+11,add(12)(q15),+13,-16,-17,+22[5]/49,sl,+13[5]/48,sl,add(17)(p11.2),del(20)(q11.2)[1]/46,XY[9].	Neg	ND	GCLAC	12.00	NR	11	12.6
39	Male	Unknown	46,XY,t(8;21)(q22;q22)[8]/45,sl,-7[11]/46,XY[1]	Neg	Neg	GCLAC	0.00	CR	1.2	4.81
51	Male	Caucasian	46,XY[20]	Neg	Neg	GCLAC	0.00	CR	10	4.2
62	Female	Caucasian	46,XX[20]	Neg	Neg	GCLAC + Cord	5.00	NR	8.57	7.24
65	Male	Caucasian	48,XY,+X,+21[20]	Neg	Neg	Tosedostat + ARAC	14.25	NR	22.2	25.9
82	Male	Caucasian	46,XY, t(5;12) with rearrangements involving 12q13	Neg	Neg	Benda + IDA	20.00	NR	13.1	7.62
36	Male	Pacific Islander	46,XY[20]	Neg	Neg	7 + 3 (DNR90)	0.00	CR	7.1	4.71
63	Female	American Indian or Alaska Native	46,XX [20]	Neg	Neg	7 + 3 (DNR60)	0.00	CR	3.85	0

53	Male	Caucasian	46,XY	Neg	Neg	IAP	0.00	CR	4.44	0
46	Female	unknown	46,XX[20]	Pos	Neg	7+3 (IDA)	2	CR	8.46	0

Supplementary Table 2. Expression of oncogenic drivers by RNA-seq in Il1rl1$^{f/f}$ vs Il1rl1$^{f/f\ Mx1Cre}$ LSCs.

Gene symbol	Il1rl1$^{f/f}$ LSC-1	Il1rl1$^{f/f}$ LSC-2	Il1rl1$^{f/f}$ LSC-3	Il1rl1$^{f/f}$ LSC-4	Il1rl1$^{f/f}$ Mx1Cre LSC-1	Il1rl1$^{f/f}$ Mx1Cre LSC-2	Il1rl1$^{f/f}$ Mx1Cre LSC-3	Il1rl1$^{f/f}$ Mx1Cre LSC-4
Tet1	0.89	0.66	0.71	0.72	0.20	0.20	0.10	0.12
Glis2	0.94	0.95	0.90	0.96	0.17	0.27	0.24	0.59
Prkch	1.09	1.10	1.16	1.06	0.66	0.56	0.58	0.85
Ptk7	1.17	1.34	1.17	1.09	0.35	0.53	0.35	0.67
Bcl2a1a	1.20	1.45	0.93	1.05	0.11	0.08	0.62	0.98
Hdac9	1.25	1.17	1.18	1.15	0.76	0.77	0.78	0.70
Nfatc2	1.41	1.37	1.41	1.44	0.86	1.02	0.90	1.01
Flt1	1.62	1.58	1.62	1.63	0.59	0.56	0.53	0.55
Gtsf1	1.67	1.60	1.77	1.53	0.91	1.00	0.99	1.00
Il1rap	1.72	1.65	1.64	1.62	1.41	1.30	1.40	1.37
Deptor	1.87	1.74	1.78	1.96	1.43	1.32	1.54	1.39
Ptprj	2.24	2.36	2.38	2.28	1.59	1.73	1.90	2.08
Braf	2.48	2.40	2.45	2.48	2.14	2.23	2.05	2.20
Dnmt3b	2.55	2.50	2.53	2.45	2.45	2.49	2.41	2.16
Pcgf5	2.63	2.64	2.66	2.63	2.22	2.17	2.21	2.04
Phf7	2.70	2.81	2.77	2.77	2.17	2.33	2.22	2.23
Nedd9	3.55	3.66	3.63	3.68	3.03	3.07	3.24	2.96
Runx2	3.62	3.55	3.55	3.68	3.16	3.29	3.33	3.32
Pdk1	3.67	3.65	3.61	3.69	3.00	2.93	2.95	2.90
Pak1	4.15	4.16	4.10	4.21	3.83	3.67	3.66	3.91
Six1	4.33	4.35	4.33	4.38	2.62	2.79	2.70	2.58
Ash2l	4.53	4.51	4.60	4.45	3.93	3.98	4.00	3.85
Aff1	4.72	4.52	4.55	4.71	3.88	3.98	4.05	3.95
Kit	4.80	4.77	4.76	4.81	4.22	4.38	4.38	4.33
Stfa2l1	5.16	5.47	5.52	5.30	2.50	2.34	2.50	0.96
Plk1	5.67	5.63	5.65	5.72	5.26	5.21	5.24	5.25
Myc	6.39	6.34	6.35	6.41	5.73	5.83	5.85	5.80
Cebpe	6.55	6.76	6.70	6.62	6.01	6.02	6.06	5.77
H1f0	6.56	6.57	6.50	6.54	5.78	5.78	5.74	5.85
Stfa1	7.29	7.36	7.48	7.33	3.65	3.75	3.70	3.20

Supplementary Table 3. Expression of cell cycle signatures by RNA-seq in Il1rl1$^{f/f}$ vs Il1rl1$^{f/f\ Mx1Cre}$ LSCs.

Gene symbol	Il1rl1$^{f/f}$ LSC-1	Il1rl1$^{f/f}$ LSC-2	Il1rl1$^{f/f}$ LSC-3	Il1rl1$^{f/f}$ LSC-4	Il1rl1$^{f/f}$ Mx1Cre LSC-1	Il1rl1$^{f/f}$ Mx1Cre LSC-2	Il1rl1$^{f/f}$ Mx1Cre LSC-3	Il1rl1$^{f/f}$ Mx1Cre LSC-4
Ep300	12.48	12.35	12.21	12.05	11.82	12.04	11.92	11.44
Myc	12.40	12.37	12.20	12.00	11.40	11.51	11.54	11.05
Ccna2	12.01	11.99	11.79	11.53	11.46	11.40	11.55	11.00
Ccnb1	11.84	11.84	11.63	11.37	11.01	10.79	10.93	10.44
Cdc20	11.79	11.81	11.60	11.40	11.06	11.00	11.08	10.59
Ccnb2	11.41	11.48	11.33	11.01	10.78	10.73	10.79	10.23
Crebbp	11.08	10.93	10.86	10.62	10.47	10.58	10.44	10.06
Cdc16	10.35	10.34	10.15	10.04	9.95	9.88	9.98	9.47
Cdk7	9.93	9.95	9.73	9.54	9.46	9.28	9.44	8.78
Chek2	9.90	10.01	9.65	9.46	9.35	9.34	9.31	8.87
Skp2	9.50	9.60	9.19	9.10	9.04	9.14	9.25	8.59
Ttk	10.57	10.46	10.32	10.14	9.87	9.78	9.91	9.32

Supplementary Table 4. Expression of metabolomic signatures by RNA-seq in Il1rl1$^{f/f}$ vs Il1rl1$^{f/f\ Mx1Cre}$ LSCs.

**Gene symbol**	Il1rl1$^{f/f}$ **LSC-1**	Il1rl1$^{f/f}$ **LSC-2**	Il1rl1$^{f/f}$ **LSC-3**	Il1rl1$^{f/f}$ **LSC-4**	Il1rl1$^{f/f}$ Mx1Cre **LSC-1**	Il1rl1$^{f/f}$ Mx1Cre **LSC-2**	Il1rl1$^{f/f}$ Mx1Cre **LSC-3**	Il1rl1$^{f/f}$ Mx1Cre **LSC-4**
Smpdl3b	0.76	0.92	0.97	0.75	0.12	0.12	0.16	0.27
Fabp4	1.07	1.09	1.04	0.78	0.09	0.13	0.21	0.81
Sirt5	1.44	1.43	1.43	1.44	0.92	1.12	1.09	0.99
Ace	1.51	1.64	1.70	1.42	0.73	0.15	0.30	0.12
Npy	1.66	1.97	1.69	1.92	0.90	1.63	1.22	1.81
Mrgpra2b	2.21	2.45	2.33	2.22	0.82	0.68	1.03	0.17
Ffar2	2.22	2.40	2.24	2.07	1.31	0.98	1.32	0.49
Pfkfb4	2.49	2.58	2.66	2.45	2.00	2.07	2.16	1.50
Enox2	2.56	2.79	2.53	2.70	2.11	2.29	2.39	2.27
Igf1r	3.27	3.15	3.22	3.22	2.48	2.61	2.64	2.23
Lrp5	3.36	3.35	3.25	3.32	2.99	2.98	3.08	2.97
B4galt4	3.65	3.68	3.75	3.73	2.69	2.66	2.76	2.54
Ptgs1	3.96	3.98	4.03	4.05	2.43	2.72	2.65	2.71
Fbp1	4.48	4.50	4.48	4.53	2.43	2.44	2.47	2.34
Padi4	4.58	4.56	4.54	4.67	3.73	3.68	3.97	3.27
Gpx3	5.23	5.23	5.28	5.14	4.84	4.84	4.70	4.95
Grk6	6.26	6.30	6.28	6.28	5.87	5.87	5.96	5.76

Supplementary Table 5. Literature references for oncogenic drivers, cell cycle and metabolomic signatures derived from comparing RNA-seq in $Il1rl1^{f/f}$ vs $Il1rl1^{f/f\ Mx1Cre}$ LSCs.

Gene ID	Reference
Six1	https://pubmed.ncbi.nlm.nih.gov/31050834/
H1f0	https://www.nature.com/articles/ncomms9489.pdf?origin=ppub
Flt1	https://ashpublications.org/blood/article/107/4/1608/133708/VEGFR-1-FLT-1-activation-modulates-acute
Stfa2l1	https://journals.plos.org/plosone/article?id=10.1371/journal.pone.0007500
Pdk1	https://www.ncbi.nlm.nih.gov/pmc/articles/PMC4008098/; https://www.sciencedirect.com/science/article/pii/S0006497119442298
Myc	https://pubmed.ncbi.nlm.nih.gov/32040550/
Ash2l	https://pubmed.ncbi.nlm.nih.gov/23239880/
Aff1	https://www.ncbi.nlm.nih.gov/pmc/articles/PMC8256865/
Pcgf5	https://www.nature.com/articles/s41467-018-03781-0
Kit	https://journals.plos.org/plosone/article?id=10.1371/journal.pone.0124241
Cebpe	https://www.ncbi.nlm.nih.gov/pmc/articles/PMC6602096/
Plk1	https://ashpublications.org/blood/article/136/Supplement%201/34/470572/A-Stemness-Based-Screen-Identifies-PLK1-Inhibitors
Nedd9	https://www.ncbi.nlm.nih.gov/pmc/articles/PMC8500976/
Phf7	https://cellandbioscience.biomedcentral.com/articles/10.1186/2045-3701-3-41
Runx2	https://pubmed.ncbi.nlm.nih.gov/19179305/
Nfatc2	https://ashpublications.org/blood/article/138/Supplement%201/3301/479217/NFATC2-regulates-Targets-of-MYC-Signaling-in-MLL
Pak1	https://www.ncbi.nlm.nih.gov/pmc/articles/PMC4551362/#:~:text=Targeting%20of%20PAK1%20inhibits%20primary,network%20of%20MYC%20target%20genes.
Braf	https://www.nature.com/articles/2403201
Tet1	https://www.ncbi.nlm.nih.gov/pmc/articles/PMC3718141/
Ptk7	https://pubmed.ncbi.nlm.nih.gov/20558616/
Hdac9	https://www.nature.com/articles/ncomms9489.pdf?origin=ppub
Deptor	https://www.ncbi.nlm.nih.gov/pmc/articles/PMC4231659/
Ptprj	https://www.ncbi.nlm.nih.gov/pmc/articles/PMC6278956/
Il1rap	https://www.ncbi.nlm.nih.gov/pmc/articles/PMC8634188/
Gtsf1	https://patentimages.storage.googleapis.com/07/09/8b/5b6e215b07976c/EP2412825A1.pdf
Prkch	https://www.ncbi.nlm.nih.gov/pmc/articles/PMC6731097/
Glis2	https://www.ncbi.nlm.nih.gov/pmc/articles/PMC7000481/

Bcl2a1a	https://pubmed.ncbi.nlm.nih.gov/23118966/
Dnmt3b	https://journals.sagepub.com/doi/full/10.1177/1177271919846454
Stfa1	https://www.nature.com/articles/s41598-019-53610-7#Sec15
Grk6	https://www.ncbi.nlm.nih.gov/pmc/articles/PMC5260904/
Gpx3	https://rupress.org/jem/article/209/5/895/41173/A-role-for-GPx3-in-activity-of-normal-and-leukemia
Lrp5	https://www.ncbi.nlm.nih.gov/pmc/articles/PMC6988868/
Sirt5	https://www.ncbi.nlm.nih.gov/pmc/articles/PMC8133360/
Enox2	https://ashpublications.org/blood/article/128/22/3059/113548/The-Cell-Surface-NADH-Oxidase-ENOX2-Is-Highly
Pfkfb4	https://pubmed.ncbi.nlm.nih.gov/32299611/
Igf1r	https://www.ncbi.nlm.nih.gov/pmc/articles/PMC3171095/; https://www.frontiersin.org/articles/10.3389/fphar.2018.00687/full
Padi4	https://www.ncbi.nlm.nih.gov/pmc/articles/PMC8486204/
B4galt4	https://www.nature.com/articles/cddis2013186
Fabp4	https://www.ncbi.nlm.nih.gov/pmc/articles/PMC5457366/
Ptgs1	https://www.ncbi.nlm.nih.gov/pmc/articles/PMC2799062/
Smpdl3b	https://www.frontiersin.org/articles/10.3389/fmolb.2021.695601/full
Ffar2	https://www.nature.com/articles/bjc2017307
Ace	https://ashpublications.org/bloodadvances/article/5/7/2012/475687/CDX2-regulates-ACE-expression-in-blood-development
Mrgpra2b	https://www.ncbi.nlm.nih.gov/pmc/articles/PMC8486204/
Fbp1	https://pubmed.ncbi.nlm.nih.gov/34363719/
Hif3a	https://www.nature.com/articles/leu201324
Ep300	https://epigeneticsandchromatin.biomedcentral.com/articles/10.1186/s13072-018-0197-x
Myc	https://www.nature.com/articles/s41598-019-54917-1
Ccna2	https://www.ncbi.nlm.nih.gov/pmc/articles/PMC556537/
Ccnb1	https://onlinelibrary.wiley.com/doi/abs/10.1002/jcp.26816
Cdc20	https://www.ncbi.nlm.nih.gov/pmc/articles/PMC5815435/
Ccnb2	https://www.sciencedirect.com/science/article/abs/pii/S1078143915005633?via%3Dihub
Crebbp	https://epigeneticsandchromatin.biomedcentral.com/articles/10.1186/s13072-018-0197-x
Cdc16	https://www.sciencedirect.com/science/article/pii/S1097276500800465
Cdk7	https://www.embopress.org/doi/full/10.1038/emboj.2012.94
Chek2	https://pubmed.ncbi.nlm.nih.gov/19151762/

Skp2	https://www.nature.com/articles/s41422-020-0372-z
Ttk	https://www.pnas.org/doi/abs/10.1073/pnas.1700234114

Supplementary Table 6. Anti-IL1RL1 neutralizing antibody doesn't not react with normal human tissues.

Human Tissues	Anti-IL1RL1 neutralizing antibody	
	1ug/ml	2ug/ml
ILEUM	0	0
SKELETAL MUSCLE	0	0
CEREBELLUM	0	0
FRONTAL LOBE	0	0
PONS	0	0
STOMACH	0	0
SPINAL CORD	0	0
PANCREAS	0	0
LIVER	0	0
LUNG	0	0
SIGMOID COLON	0	0
SPLEEN	0	0
THYROID	0	0
KIDNEY	0	0

Supplementary Table 7. Patient demographics and sample characteristics of AML PDX models

PDX	Age (years)/ Sex	Race/ Ethnicity	Sample collected at	Karyotype (patients marrow)	Oncofusion
NTPL-377	1.5/F	Hispanic	Diagnosis	46, XX, t(9;11)(p21;q23)[20]	MLL-AF9 (KMT2A-MLLT3)
NTPL-477	19/M	Caucasian	Relapse	46, XY[23] / del(9)(q13q22) and del(12)(p11.2p13)[3]	MLL-FLNB (KMT2A-FLNB)
NEM9	15/F	Black	Diagnosis	46,XX,t(6;11)(q27;q23)[17]/46,XX[4]	MLL-AF6 (KMT2A-MLLT4)
NEM10	7/F	White	Diagnosis	46,XX,t(10;11)(p11.2;q23)[20]	MLL-AF10 (KMT2A-MLLT10)
DF-2	1/M	Caucasian	Relapse	46,XY,inv(6)(q23q27)[20]	MLL-AF6 (KMT2A-MLLT4)
DF-5	15/F	Unknown	Relapse	46,XX,inv(10)(p12.2q21.2)[20]	MLL-AF10 (KMT2A-MLLT10)
NTPL-301	13/F	Unknown	Secondary neoplasm	42~43, X, t(2;16)(q21;p13.1),add(4)(q21),der(5)t(5;12)(q13;q11.2),-7,add(12)(p11.2),add(15)(q22,-17,-19,add(20)(p13),+1~2mar[cp8/42,sl,-13[cp5]/42,sdl1,+del(13)(q12q14),-add(20)[2]/42,sdl2,-der(5),+add(7)(q22), ins(10)9p11.2)[2]/42,sl2,-7,der(13)t(7;13)(q11.2;p11.2)[2]/45,X,-X[1]	None
NTPL-511	14/M	Unknown	Diagnosis	47, XY,+8[1]/46,XY[29	NUP98-NSD1
