# Supplementary material for: Dual targeting of tumoral cells and immune microenvironment by blocking the IL-33/IL1RL1 pathway
Source: Nat Commun. 2025 Jul 14;16:6369. doi: 10.1038/s41467-025-61567-7 (PMC12259856; doi:10.1038/s41467-025-61567-7)
Supplement: Supplementary file 2 — Description of Additional Supplementary Files [file 41467_2025_61567_MOESM2_ESM.pdf]

Description of Additional Supplementary Files

Supplementary Data 1. Differentially expressed genes comparing RNA-seq in Il1rl1f/f vs Il1rl1f/f Mx1Cre LSCs (adjusted p-value < 0.05 and absolute fold change > 1)
